# Do the Eyes Have It? A Systematic Review on the Role of Eye Gaze in Infant Language Development

**DOI:** 10.3389/fpsyg.2020.589096

**Published:** 2021-01-08

**Authors:** Melis Çetinçelik, Caroline F. Rowland, Tineke M. Snijders

**Affiliations:** ^1^Language Development Department, Max Planck Institute for Psycholinguistics, Nijmegen, Netherlands; ^2^Donders Institute for Brain, Cognition and Behaviour, Radboud University, Nijmegen, Netherlands

**Keywords:** eye contact, gaze following, language development, word acquisition, object processing

## Abstract

Eye gaze is a ubiquitous cue in child–caregiver interactions, and infants are highly attentive to eye gaze from very early on. However, the question of why infants show gaze-sensitive behavior, and what role this sensitivity to gaze plays in their language development, is not yet well-understood. To gain a better understanding of the role of eye gaze in infants' language learning, we conducted a broad systematic review of the developmental literature for all studies that investigate the role of eye gaze in infants' language development. Across 77 peer-reviewed articles containing data from typically developing human infants (0–24 months) in the domain of language development, we identified two broad themes. The first tracked the effect of eye gaze on four developmental domains: (1) vocabulary development, (2) word–object mapping, (3) object processing, and (4) speech processing. Overall, there is considerable evidence that infants learn more about objects and are more likely to form word–object mappings in the presence of eye gaze cues, both of which are necessary for learning words. In addition, there is good evidence for longitudinal relationships between infants' gaze following abilities and later receptive and expressive vocabulary. However, many domains (e.g., speech processing) are understudied; further work is needed to decide whether gaze effects are specific to tasks, such as word–object mapping or whether they reflect a general learning enhancement mechanism. The second theme explored the reasons why eye gaze might be facilitative for learning, addressing the question of whether eye gaze is treated by infants as a specialized socio-cognitive cue. We concluded that the balance of evidence supports the idea that eye gaze facilitates infants' learning by enhancing their arousal, memory, and attentional capacities to a greater extent than other low-level attentional cues. However, as yet, there are too few studies that directly compare the effect of eye gaze cues and non-social, attentional cues for strong conclusions to be drawn. We also suggest that there might be a developmental effect, with eye gaze, over the course of the first 2 years of life, developing into a truly ostensive cue that enhances language learning across the board.

## Introduction

Social interaction plays a critical role in language acquisition. Children typically learn language through face-to-face interactions with their caregivers in social contexts, and face-to-face communication is inherently multimodal. The communicating social partners exchange a variety of information beyond the verbal domain, using facial expressions, gestures, and most pertinently for the present paper, eye gaze.

Eye gaze is, in fact, a central element in human communication. Gaze cues during a communicative interaction can indicate social engagement, reflect a desire to communicate, reveal the speaker, and the listener's goals and feelings, and can direct the attention of the listener to objects in the environment (Kleinke, 1986). Eye gaze can act as an ostensive cue to a speakers' intent, by specifying the addressee of the communication and signaling that the accompanying actions are communicative and meaningful rather than random acts (Csibra, 2010). This last function is especially crucial for human infants, since their limited knowledge of language means that they cannot rely on the semantic context of the speech signal to understand that communication is directed toward them. Rather, they can infer that the social partner (i.e., the adult) is addressing them by social signals in communication, such as eye gaze, infant-directed speech, and calling the infant's name (Csibra and Gergely, 2009). The current review focuses specifically on the role of eye gaze in infant language development, over and above other social cues.

Not only do adults often use such social cues when communicating with infants, but infants also show a sensitivity to, and preference for, these signals from early on. Infants display a sensitivity to eye gaze in at least two distinct ways. First, they engage in mutual eye contact with their social partner. Newborns look longer at faces with open eyes than faces with closed eyes (Batki et al., 2000). They also prefer faces with direct gaze with which they can engage in mutual eye contact, as evidenced by their preference for direct gaze only for upright and not for inverted faces (Farroni et al., 2002, 2004). This ability to detect and engage in mutual eye contact in live socially interactive settings develops further over the first 4 months of life (Vecera and Johnson, 1995). Second, infants learn to follow an interlocutor's gaze. Infants begin developing gaze-following abilities between 2 and 4 months, which become fairly stable by 6–8 months (D'Entremont et al., 1997; Gredeback et al., 2010). Orienting to gaze cues becomes almost automatic, with adult's gaze direction causing fast visual attention shifts even in infants as young as 3 months of age (Hood et al., 1998).

However, it is not yet clear to what extent infants' sensitivity to social eye gaze has a function beyond basic perception/attention. In particular, it is not yet clear whether, and in what ways, it also facilitates infants' learning in cognitive domains, such as language. There are (at least) two reasons to expect that a sensitivity to eye gaze might facilitate language development. Acting as an ostensive cue, mutual eye gaze (i.e., eye contact) can convey the communicative intent of the caregiver and can put infants in a highly receptive state for accompanying or upcoming information (Csibra and Gergely, 2009). This is the role of eye gaze according to natural pedagogy theory (Csibra and Gergely, 2009), which holds that ostensive cues, such as eye gaze have a special status in human ontogeny. On this theory, human communication creates opportunities for a transfer of knowledge between a sender and a receiver (caregiver and infant, in this case), and these opportunities are marked by an abundance of ostensive cues, such as eye gaze. Human infants are argued to be innately specified to be sensitive to such cues, such that the presence of those cues puts them in a highly receptive state for upcoming or accompanying stimuli.

Yet, eye gaze could also act as a more basic, simple, attentional cue. In particular, sensitivity to eye gaze could allow infants to optimize the use of limited attentional resources, by directing attention to only those parts of the environment in which the other partner is interested (Niedzwiecka et al., 2018). This could be in the form of mutual gaze or gaze following. Mutual gaze draws infants' attention to the social partner and presumably to the speech signal provided by them. Gaze following directs their attention to a target location in the environment, which facilitates the learning of object properties and their names (Wu et al., 2014). On this view, eye gaze has no special social status but is simply an attentional cue. It may not be different than other low-level cues, such as movement, which equally attract infants' attention. Eye gaze provides learning opportunities for infants through attention modulation rather than serving a special communicative purpose (Szufnarowska et al., 2014).

A related issue concerns the types of language learning tasks that are facilitated by eye gaze. Most studies to date have focused on the role of gaze following in learning about objects in the environment, studies in which children follow the gaze of an interlocutor toward an object and which then test whether gaze following facilitates the encoding of object properties or object–word mappings. However, it is possible that eye gaze might have a more general learning enhancement function, as specified by the natural pedagogy theory. In this case, we might expect eye gaze to have a facilitatory effect on other language tasks (e.g., learning to process speech).

The goal of this study was thus to systematically review the literature on the role of eye gaze in early language learning in the first 2 years of life (infancy). Given the focus on infancy, the scope of the review is mainly restricted to vocabulary development (eye gaze may play a role in other areas, such as grammar and pragmatics, but these develop later in childhood). The literature searching process identified relevant work not only on vocabulary development itself but also in three subdomains that are crucial for the development of vocabulary: word–referent mapping (labeling), object processing, and speech processing. We summarize work in all four domains below, before turning to the question of why eye gaze may facilitate language learning. In particular, we discuss whether there is evidence that eye gaze is a highly specialized socio-cognitive cue that puts infants in a highly charged receptive learning state, as specified by the natural pedagogy theory, or whether it is simply a highly effective attentional cue.

## Method

We searched the PsycInfo, PubMed, Scopus, and Web of Science databases from the beginning of database records until January 2019. In order to capture the existing literature, we used broad search terms, infan^*^ AND (eye contact OR gaze) AND (attention OR learning), within the title or the abstract. We focused on “attention” and “learning,” rather than narrowing down to language, because we wanted to include, at this first step, papers that assessed the role of eye gaze in aspects of cognitive development that were relevant to language learning, such as the ability to learn to identify objects in the environment, crucial for object labeling. This search yielded 2,061 papers in total, which was reduced to 1,405 entries after duplicate removal.

We then narrowed the search to the following inclusion criteria: (1) peer-reviewed articles written in English, which (2) study typically developing human infants between 0 and 24 months of age, and (3) present data for a group of participants (that meet the second inclusion criteria) in the domain of cognitive development/learning. We excluded papers that simply documented the development of infant eye gaze behavior without addressing the effect of such abilities on learning, papers that investigated the role of eye gaze in socio-emotional development (e.g., understanding of facial emotional expression) and motor development, and papers that investigated the role of different types of cue on infant sensitivity to eye gaze (e.g., infant temperament, maternal depression) where such studies did not also include an element of learning or processing. We also excluded papers investigating eye gaze behaviors in children with autism or other developmental disabilities, as these focused on different questions (e.g., how to characterize the socio-cognitive abilities of children with autism).

**Figure 1:**
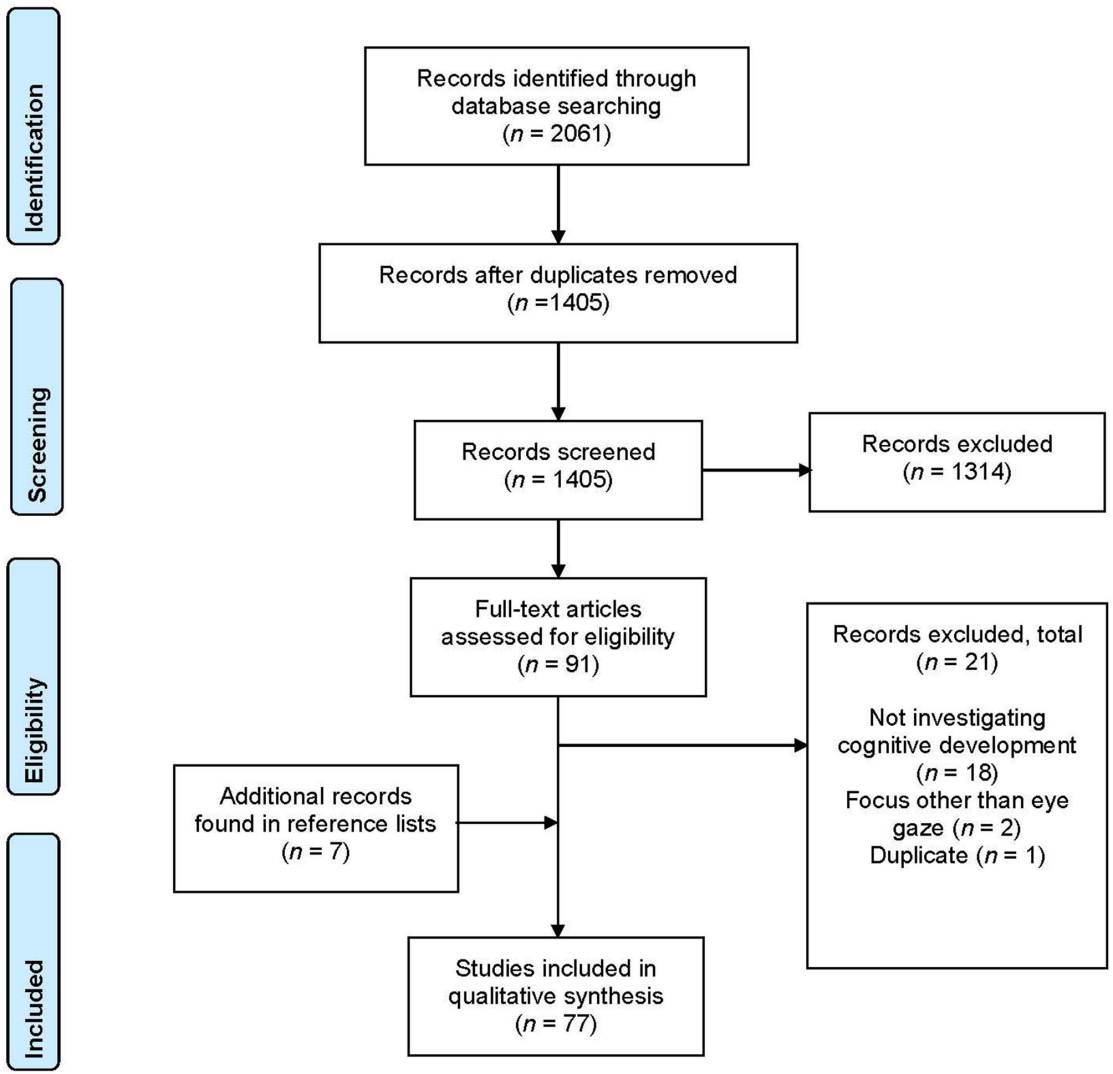
Preferred Reporting Items for Systematic Reviews and Meta-analyses (PRISMA) flowchart of the literature search, following Moher et al. (2009).

We first screened the 1,405 entries based on their titles and abstracts with regards to the inclusion criteria. We identified 91 papers as eligible for full-text review. An additional seven papers were identified through hand searching the reference lists of the retained articles and were added to the review, resulting in 98 papers. In the second stage, we retrieved the full text of each paper and reviewed them for inclusion, which resulted in 77 papers included in the final review. Figure 1 illustrates the literature search process.

## Results

The papers identified in the review can be broadly divided into two main themes. The first set of papers documented the effect of eye gaze on four developmental domains: the effect on (1) vocabulary development in general, and then, its effect in three subdomains that are crucial for the development of vocabulary; (2) word–referent mapping (labeling); (3) object processing; and (4) speech processing. The second set discussed theories that aim to explain why eye gaze might be facilitative for learning. We discuss each here in turn. Supplementary Table 1 summarizes the main information of all studies included.

### Effects of Eye Gaze on Infant Learning

#### Vocabulary Development: Do Infants With Better Joint Attention Skills Later Develop Better Vocabulary?

Joint attention refers to the caregiver–infant dyads' shared attention to an object or an event while maintaining their attention to each other. It encompasses a set of socio-cognitive skills that develop in the first year of life, such as pointing, referencing, and gaze following. Gaze following is of utmost importance in studying joint attention in the context of language development, as most of the studies that investigated links between joint attention and vocabulary development use children's gaze direction and following as a measure of the understanding of the shared (joint) attentional focus (Akhtar and Gernsbacher, 2007). Joint attention involves establishing mutual eye contact with the social partner and then alternating gaze between the partner and the object (either in the presence or absence of other cues, such as pointing or verbal referencing).

Gaze following is an important social skill that develops in the first year of life. Following the gaze of an interactional partner enables infants to engage in joint attentional periods with that partner, and this provides potential learning opportunities for the young language learner. By engaging in joint attention with the adult and following their gaze, infants can selectively attend to a single source (e.g., an object). Hereby, they can direct their attentional resources (which are otherwise naturally limited) to the properties of that object, allowing them to disambiguate the speaker's likely referent. Thus, the ability to follow a partner's gaze, and engage in joint attention, is likely to promote vocabulary development.

Our review identified 17 studies that link gaze following and vocabulary development. In most of these studies, children's understanding of the shared (joint) attentional focus was measured by testing whether the child followed the adult gaze to a target location or object when the adult turned toward the target and then whether they alternated their gaze between the adult and the target. To do this, many studies used standardized measures, such as the Early Social Communication Scales (Mundy et al., 2003a) that assess children's non-verbal communication skills, including initiating and responding to joint attention. Subsequent vocabulary growth was assessed by parental reports using standardized language tests (such as the MacArthur-Bates Communicative Developmental Inventories; Fenson et al., 2007) at later ages, with the respective subtests for comprehensive and productive vocabulary, and in some studies, complemented with laboratory observation data using standardized language measures. As far as possible, we discuss relations with receptive and expressive vocabulary separately, but there is some overlap here, as the reviewed papers often test both and do not always distinguish between them.

Most of the studies reviewed identified positive links between joint attentional periods between infants and parents and infants' later vocabulary development (Carpenter et al., 1998; Morales et al., 1998; Brooks and Meltzoff, 2005, 2015; Beuker et al., 2013). In terms of receptive vocabulary, individual differences in responding to joint attention, indexed by infants' gaze following scores, were found to be meaningful at as early as 6 months and contributed to differences in receptive vocabulary scores at 12 months (Morales et al., 2000b). Gaze-following behavior at 10–11 months predicted receptive vocabulary at both 14 and 18 months (Brooks and Meltzoff, 2005). Further, full-term infants' responsiveness to gaze alternations in triadic interactions at 9 months and initiating triadic interactions at 14 months were positively correlated with later language, such that infants with more responsivity to gaze shifts had better receptive and expressive language scores at 30 months (De Schuymer et al., 2011). One study, however, did not find a link between infants' responding to joint attention skills at 12 months and their receptive vocabulary at 12 and 18 months, although it did report correlations with expressive vocabulary at 18, 21, and 24 months (Markus et al., 2000).

Many studies also found infants' joint attentional skills to be predictive of expressive vocabulary when tested at later ages. Studies have reported that (1) individual differences in infants' gaze following abilities at 6 months were positively linked to their receptive vocabulary at 12 months and subsequent expressive vocabulary at 18, 21, and 24 months (Morales et al., 1998); (2) infants' responding to joint attention at 6, 8, 10, 12, and 18 months was positively correlated with expressive vocabulary size at 30 months (although individual differences at 21 and 24 months did not predict language development; Morales et al., 2000a); (3) individual differences in responding to joint attention at 9 and 12 months and initiating joint attention at 18 months predicted 24-months expressive language (Mundy et al., 2007); (4) responding to joint attention at 14 months predicted 24-months expressive language, when controlling for general cognitive development (Mundy et al., 2003b); and that (5) responding to joint attention at 16 months was related to receptive language at the time of test and both later receptive and expressive vocabulary (Mundy and Gomes, 1998). Brooks and Meltzoff reported that infants with higher gaze-following scores at 10.5 months were able to produce more mental-state words at 2.5 years, reflecting an effect both on their vocabulary and theory of mind capacities (Brooks and Meltzoff, 2015). One study did not report a significant link: Morales et al. (2000b) did not find links between gaze-following scores at 6 months and expressive vocabulary at 12 months, but note that this is perhaps not surprising since there is very little variation in expressive vocabulary at 12 months. Overall, then, we conclude that the literature overwhelmingly supports the idea of a meaningful relationship between joint attentional abilities and receptive and expressive vocabulary development in infancy.

Interestingly, the findings of one study (Tenenbaum et al., 2015) suggest that eye gaze cues might enhance learning by focusing infants' attention to linguistically relevant information as well as to their referents in the environment. This study tested 12-months-old infants' gaze following to an object at the point at which the adult was describing the object, as well as their looking toward the speaker's mouth, rather than simply testing gaze following ability *per se*. Infants' gaze performance in this task predicted later expressive vocabulary at 18 and 24 months. These findings are in line with the literature suggesting a developmental shift in infants' attention from the speaker's eyes to their mouth between 4 and 8 months, with a shift back to eyes starting to emerge around 12 months (Lewkowicz and Hansen-Tift, 2012).

On a related note, some studies suggest that later language development is better predicted when we consider multiple pre-linguistic communicative behaviors together, rather than focusing only on gaze following. Brooks and Meltzoff (2008), using growth curve modeling, reported that, together, pointing, gaze following, and looking time (duration of looks at the target object) at 10–11 months predicted more of the variance in the speed of vocabulary growth at 2 years than the individual predictors alone. Importantly, infants' pointing and gaze-following scores did not correlate significantly with each other, suggesting that they are tapping different abilities (but also see Carpenter et al., 1998). This finding suggests that different pre-linguistic abilities may serve different functions; for example, while pointing helps infants initiate triadic attention with the parent, following the parent's gaze may enable infants to understand the referent of words. In a similar vein, D'Odorico et al. (1997) reported that the coordinated development of two communicative behaviors between 12 and 20 months, gaze and vocalizations, was a sign of conversational competence, which then predicted language production at 20 months. Although care must be taken when interpreting this result as the sample size was small (*N* = 13), the findings are supported by recent studies that suggest a key role of infant vocalizations in the pre-linguistic period (McGillion et al., 2017; Donnellan et al., 2020). Interestingly, coordinated gaze-vocalization behavior at 11 months, which may signal infants' communicative intent, was the strongest predictor of expressive vocabulary at multiple time points up to 24 months (Donnellan et al., 2020).

However, while many studies reported positive relationships between gaze following or gaze alternation skills and language development, some have suggested that these effects are mediated by other factors. For instance, a recent study measuring both parent–infant joint attention and infant sustained attention in naturalistic interactions found that both joint attention and infant sustained attention at 9 months predicted language development at 12 and 15 months, but joint attention by itself was not a strong predictor (Yu et al., 2019). Joint attention periods that did not coincide with sustained infant attention to the target object did not predict later vocabulary scores, while infant sustained attention to the object did, regardless of the joint attentional state at the time of the naming event (Yu et al., 2019). Further, one study did not find any links between joint attention abilities, indexed by gaze alternation between the adult and object, at 20 months and later language, although it reported associations with theory of mind abilities (note though that the sample size was low: 13 infants; Charman et al., 2000).

In summary, with a few exceptions, the results generally support a positive association between joint attention skills and vocabulary development. However, it is not possible to directly establish cause and effect from such studies, since they are observational, and in the main, correlational. In the next three sections, we review the evidence from three subdomains linked to vocabulary acquisition that might help explain why there is such a positive association: evidence that eye gaze (a) facilitates the learning of word–object mappings, (b) facilitates the learning of object properties, and (c) facilitates speech processing.

#### Word–Object Mapping

In joint attentional contexts, adult and infant attend to the same object while maintaining shared attention with each other, established by mutual gaze (Carpenter et al., 1998). In such instances, eye gaze can direct infants' attention to a specific object in their environment, thereby creating an ideal window for them to learn more about properties of that object, including the label used to refer to it. Hence, gaze following can be a reliable strategy for infants to map words onto objects. In this section, we review evidence for the role of gaze (following) in forming these word–object mappings, based on the 12 papers identified by our systematic review.

As early as 6–8 months of age, the frequency of infants' gaze switches between their mother and an object that occur just at the point at which the mother names an object can affect word learning; two studies reported that infants who switched their gaze frequently were more likely to learn word–object mappings in naturalistic interactions (Gogate et al., 2006; Matatyaho and Gogate, 2008). However, evidence for a sophisticated ability to use gaze cues to form word–object associations does not seem to appear until the second year of life. In particular, the evidence suggests that it is only toward the end of the second year that infants can use gaze cues to map labels onto the object in situations where perceptual salience cues conflict with social (eye gaze) cues (Moore et al., 1999). For instance, Moore et al. (1999) demonstrated that, when presented with a novel label, 24-months-old, but not 18-months-old, picked the toy to which the adult had directed their gaze during familiarization. This was true even when the saliency of the other object was higher (salience was manipulated by illuminating and rotating one of the two possible referents of the label). Eighteen-months-old infants only correctly matched the object to its label when both referential and salience cues reference the same object (Moore et al., 1999). Similarly, Hollich et al. (2000) reported that 24-months-old infants chose to follow adult's gaze direction to map words to objects, rather than using other salient but non-referential cues.

It is possible that younger children can use eye gaze cues to learn word–object associations but only in certain situations. In one study, infants aged between 12 and 18 months followed the gaze of a stranger as well as that of their caregiver but only formed word–object associations when following the caregiver's gaze (Barry-Anwar et al., 2017), although note that in other studies, infants of this age did learn from a stranger (e.g., Moore et al., 1999; Hirotani et al., 2009). The “social” nature of the agent also seems to be important for younger children to establish word–object mappings; 18-months-old learned the names of novel objects when the labels were provided by a human, but not a robot, although they did follow the robot's gaze (O'Connell et al., 2009). Even older infants' ability to learn from eye gaze cues can be derailed. For example, Graham et al. (2010) reported that 24-months-old infants' learning from gaze cues was also affected by “default” biases, such as mutual exclusivity (the assumption that a single object has only one label), with infants relying on mutual exclusivity when gaze cues offered conflicting information. Optimal learning only occurred when eye gaze and mutual exclusivity provided coinciding information (Graham et al., 2010).

There also seems to be some evidence that infants only treat gaze cues as referential cues (i.e., cues to object labels rather than simply low-level attentional cues) from about 24 months of age. Graham et al. (2011) tested 24-months-old infants' word learning both when the experimenter's gaze cued the location of the object and when the gaze cued the object itself. In the location condition, the experimenter looked at the target location, provided the label, and then placed the object in that location. In the object condition, the object was presented in the target location before the label was given. The results showed that, although infants followed gaze equally well in both conditions, they formed word–object mappings only when the experimenter's gaze cued the object already in the location (the object condition). The authors argued that this was because the infants treated gaze as a signal of referential intent; they expected to find an object to which the word can be mapped in the cued-at location and only learned the label in the condition in which this expectation was satisfied. Interestingly, one study suggests that even infants' preferred social cue preferences may change with age: Paulus and Fikkert (2014) reported that young infants (14-months-old) relied more on eye gaze cues when learning word–object mappings, but adults and older infants (24-months-old) relied more on pointing cues.

In summary, it seems that eye gaze influences infants' learning of word–object pairings. There exists a clear developmental trend in increasing sophistication over the first 2 years of life, supported by infants' developing attention, memory, and information processing capacities (Yurovsky and Frank, 2017). However, it is not always clear from such studies whether it is eye gaze *per se* that is driving the effect because it is usually difficult to disentangle eye gaze and other joint attentional cues. For instance, Hirotani et al. (2009) used an event-related potential (ERP) paradigm to investigate the effects of joint attention on infants' word learning at 18–21 months. Infants were taught novel word–object combinations in either a joint attention context (eye contact, positive tone of voice) or non-joint attention context (eye contact averted, neutral tone of voice). For words learned in the joint attention context, incongruent object–word pairs resulted in a late negativity, similar to a N400 effect, reflecting a failure in semantic integration. However, as both eye gaze and other social cues, such as the speaker's tone of voice were jointly manipulated, it is difficult to conclude which cue drove the effect.

There is one study, however, that provides evidence that infants can use eye gaze cues under more tightly controlled experimental settings. Houston-Price et al. (2006) used prerecorded videos of an experimenter turning her head (with gaze following) to one of the two objects placed on her right and left, while a label was provided over the loudspeaker. The use of a prerecorded video allowed the authors to control for the presence of other (covert) joint attentional cues. Fifteen-months-old successfully learned the word–object pairings in this context. Although this is only one study that needs to be replicated, such findings in controlled settings suggest that learning can indeed be driven by the presence of the intended social cue (gaze direction) and is not a result of additional, covert social cues that might occur when infants are interacting with a live experimenter (Houston-Price et al., 2006).

#### Object Processing

Another explanation for why infants' sensitivity to gaze cues might affect vocabulary development concerns the role of gaze cues in object processing. Given that a necessary precursor to learning to map words onto objects is learning to identify objects themselves, it may be that eye gaze facilitates object processing directly, which then indirectly facilitates word learning. By 4 months, infants start to follow an adult's gaze to a location and use this cue to switch their attention to that location. It has been suggested that this facilitates processing of the properties of the target object rather than other competing stimuli in the environment (Reid and Striano, 2005). In other words, infants not only are more likely to pay attention to a specific object as a result of the adult's gaze direction but also will be more likely to process that object and thus learn more about it. This facilitatory effect is likely to contribute to infant's ability to form word–object mappings by enhancing successful encoding of object properties, to which words are then mapped, and thus language development. In fact, object processing has been found to be a mediator of the relationship between gaze following and later vocabulary development (Okumura et al., 2017).

Our systematic review identified a large number of studies (*n* = 28) demonstrating a role for eye gaze cueing in infants' object processing. Many of these (*n* = 14) used variations on the behavioral novelty preference paradigm. In this paradigm, infants are first familiarized with novel objects using simple, prerecorded images or videos that either depict a person looking toward (cued) or away from the object (uncued) or that depict a person first establishing eye contact and then turning their head and gaze to one of two objects placed on either side of the face. In a subsequent test phase, the object(s) are presented without the face, and infant looking times to the object(s) are measured. Differences in infants' novelty preference in the test phase is taken to reflect differential processing of cued and uncued objects in the familiarization phase, possibly due to more attention to the cued object. The assumption here is that the previously uncued object will be perceived as more novel, thus resulting in longer looking times due to infants' novelty preference. In general, such studies have reported the expected novelty preference for the uncued object (see, e.g., Reid and Striano, 2005, who found this effect with 4-months-old). Similarly, social cues, such as the face and eyes turning toward the target object have been shown to enable 9-months-old infants in making inferences about object properties, even when distracting cues are present (Wu et al., 2011).

In an experimental setting using live joint attention interactions, joint attention has been shown to facilitate object processing for 9-months-old, but not 4-months-old, compared to a non-joint attentional condition in which the experimenter alternated gaze between object and the ceiling (Cleveland and Striano, 2007). A similar study found effects for 7-months-old but not 5-months-old (Cleveland et al., 2007). These results point at a developmental shift during the first year in how infants understand and make use of eye gaze cues in joint attentional settings to learn about objects. This shift occurs at about the same time as, or slightly earlier than the time that, children are starting to learn to understand, and perhaps even produce, their first word. Although 4–5-months-old infants might not be able to process the triadic interaction facilitated by eye gaze in complex interactional settings, it looks like there is a gradual shift toward more sophisticated understanding of joint attentional cueing, starting at 5 months (Cleveland et al., 2007).

However, infants' reliance on joint attention in object-processing tasks may be affected quite substantially by the nature, and in particular complexity, of the task. For example, Striano et al. (2006a) compared infant looking times in two conditions: (a) one in which the experimenter alternated their gaze between a toy and the infant while speaking about the toy during familiarization and (b) one in which the experimenter switched their gaze between a spot on the ceiling and the toy, without looking at the infant during familiarization. In the test phase, when the familiar and a novel toy were presented together, 12-months-old infants looked equally at the novel toy irrespective of the presentation condition, whereas 9-months-old looked at the novel toy only after the joint attention condition. This suggests perhaps that the reliance of infants on social cues depends on how challenging the task is at the developmental stage they are in. Object processing may be a challenging task for 9-months-old infants, who may thus rely on the presence of joint attentional cues that simplify the task by directing their attention to a specific location. By 12 months, infants may be able to parse more of their environment more easily and thus may no longer depend on such cues for simple tasks, such as processing basic properties of a single object. However, they may still heavily make use of joint attentional cues in more complex, cognitively demanding settings, such as in the presence of multiple objects, more challenging object properties, or multimodal input.

That said, infants seem to clearly understand the referential nature of the gaze following action by 12 months. Twelve- and 18-months-old infants can follow an experimenter's gaze behind barriers where an object is hidden (Moll and Tomasello, 2004). Similarly, 8- and 12-months-old show surprise reactions when objects are not at the expected location when the barrier is removed (as indicated by longer looking times; Csibra and Volein, 2008). This suggests that they expect gaze to convey information about the object's location (i.e., referential information). Further evidence that infants expect human eye gaze to convey referential information comes from studies comparing infants' reactions to human and robot gaze. In a study comparing infants' gaze following behavior of a human and a robot agent, 12-months-old infants reliably followed both human and robot gaze, but they demonstrated reliable prediction of an object at the target location only when it was cued by human gaze (Okumura et al., 2013b). Moreover, their learning about objects seemed to be affected by the humanness of their interlocutor, as they only showed enhanced processing of the object when it was gazed at by a human agent (Okumura et al., 2013a). Interestingly, children only 2 months younger, at 10th month of age, failed to predict the appearance of the object when cued by a robot or a human agent (Okumura et al., 2013b; note that although this finding may seem to contradict previous studies showing learning of object properties after gaze following in younger infants, in fact the task in this study was more challenging, as the infants had to anticipate the location of the object in order to show successful learning).

The literature reviewed above suggests a role for eye gaze cueing in facilitating infants' object processing that develops during the first year of life; infant's novelty preference for uncued objects in the test phase is taken to reflect enhanced processing of cued objects during familiarization. However, this might not have long-term learning effects. For example, in one study, although 12-months-old infants followed the experimenter's gaze to the cued object, they only displayed a novelty preference for the uncued item during the first test trial, and not during later trials (Theuring et al., 2007). This raises the possibility that gaze cueing may have only short-term effects on 12-months-old infants' processing of objects (Reid and Striano, 2005).

It is also important to note that infants might learn from non-social cues as well. Barry et al. (2015) reported that 9-months-old successfully used both social (a person's eye gaze) and non-social (a rectangle) cues to learn about statistical object regularities. However, recent electroencephalogram (EEG) studies with 4-months-old infants comparing the effect of social and non-social cues in learning object properties provided contrasting evidence, suggesting that infants' object processing was facilitated specifically by social cues (Wahl et al., 2013, 2019; Michel et al., 2019).

Another intriguing finding is that the presence of mutual gaze, possibly signaling communicative intent, might affect what infants learn about an object. Yoon et al. (2008) showed 9-months-old infants videos of an adult that either (a) pointed at an object while making eye contact with the infants and greeting them in an infant-directed manner (communicative context) or (b) reached for an object, without making eye contact or addressing the infants (non-communicative context). Infants retained information about the object identity, but not its location, when objects were presented in a communicative social context, and retained location but not identity information in the non-communicative setting. The authors suggested that ostensive cues in communication, such as eye contact, pointing, and infant-directed speech, may bias infants to encode generalizable features that support learning about object kinds. Variable information, such as the spatiotemporal features of an object, is deemed non-generalizable and thus is not retained. Note that a conceptual replication study by Okumura et al. (2016) only partially replicated the results. The authors reported an object identity bias in the communicative context but no location bias in the non-communicative context. Thus, the hypothesis that eye gaze might modulate what infants learn from interactions remains an important issue for future research.

In summary, a large number of studies report a facilitative role for eye gaze in infants' object processing. However, it is important to note that most, if not all, of the studies described above provide only indirect evidence that infants detect differences between cued and uncued objects because they are reliant on interpreting novelty preferences (they interpret novelty preference to uncued objects as indicating greater stimulus encoding or processing of the object presented in a prior cued phase). Neuroimaging studies, however, can provide more unambiguous evidence for differential processing in eye gaze cue vs. other conditions. Our review identified a number of studies (*n* = 14) that use neuroimaging paradigms to address these issues. The focus of these studies is mainly on identifying the neural mechanisms that underlie infants' enhanced processing and learning to understand why infants learn better in the presence of eye gaze cues, which we will review in detail in *Why Does Eye Gaze Facilitate Learning?* below. However, it is worth noting that a number of these studies provide direct evidence for the role of eye gaze during object processing, as they show that infants' neural responses to objects differ as a function of eye gaze during the object familiarization or test period (Reid et al., 2004; Reid and Striano, 2005; Striano et al., 2006b; Hoehl et al., 2008b, 2012; Parise et al., 2008; Kopp and Lindenberger, 2011, 2012; Wahl et al., 2013, 2019; Hutman et al., 2016; Michel et al., 2019). Thus, we conclude that the balance of evidence suggests that infants can reliably use adults' gaze to facilitate attention to a location by 4–5 months of age. Gaze cues also seem to lead to enhanced object processing in infants as young as 4 months. However, again, care must be taken in interpreting the overall results, as many studies may conflate eye gaze with other ostensive, joint attentional cues.

#### Speech Processing

The review identified a small number of studies (*n* = 3) showing that the direction of eye gaze, signaling whether the infant is addressed as the receiver of the communication, also modulated infants' neural responses to speech. In one study, mutual gaze (direct vs. averted) as well as object-directed gaze (referential vs. averted) influenced the ERP response to forward compared to backward speech in 4–5-months-old infants, both at early stages of processing (the Nc, for mutual gaze only) and at later latencies (slow wave, for both mutual and referential gaze; Parise et al., 2011). Similarly, a functional near-infrared spectroscopy (fNIRS) study by Lloyd-Fox et al. (2015) that used a naturalistic interaction design revealed that 6-months-old infants' cortical responses to infant-directed speech (and gestures) were enhanced in inferior frontal, anterior temporal, and temporoparietal regions when speech was presented with direct eye contact. These regions were found to be involved in the processing of communicative cues in previous studies (Grossmann et al., 2008). Interestingly, in this study, the facilitatory effect of eye gaze was only observed in combination with infant-directed speech, which is not surprising since this is the register caregivers generally use when talking to their infants (and which also may be processed as an ostensive cue). Besides these two studies reporting gaze effects on the neural processing of speech, one study also reported gaze effects on the discrimination of phonemic boundaries from speech. Conboy et al. (2015) examined 9.5–10.5-months-old English infants' joint attention with Spanish-speaking interlocutors in a live interactive setting in which the interlocutor described objects to the infants and read picture books to them. They found that infants' gaze shifts between the objects and the interlocutor, an index of their joint attention, predicted their perception of Spanish phonemes when tested at 11 months, such that infants with greater gaze shifts showed better neural discrimination of Spanish phoneme contrasts.

These findings suggest that eye gaze cues provided by the social partner, as well as the degree to which infants make use of them, might influence how infants process and learn from speech. In particular, gaze shifts may reflect infants' information processing abilities and signal attention to the information provided by the social partner, thereby increasing the opportunities for learning. However, as these conclusions come from only three studies, further work is needed to understand what drives this learning effect and the mechanisms that support a connection between social behavior and speech perception.

#### Why Does Eye Gaze Facilitate Learning?

In the previous sections, we presented evidence showing that infants tend to learn more in the presence of gaze cues compared to the absence of such cues. However, the discussion so far has not provided an answer to why infants learn better in the presence of gaze cues. Our systematic review process identified 32 studies that either directly addressed this question or that present evidence that speaks to this question. In this section, we first discuss the neurocognitive mechanisms by which eye gaze might have a facilitatory effect in infant's language learning. Then, we present the evidence for the different theories that aim to specify the status of eye gaze in infants' learning: do infants learn better simply because gaze is an attention-grabbing cue or does gaze hold special meaning for infants, signaling the referential and communicative intent of the adult?

##### Neurocognitive mechanisms of the facilitatory role of eye gaze

A number of the studies identified in our review demonstrated an early specialization of the cortical regions that are involved in the processing of face-to-face communication cues, such as eye gaze perception, showing that even very young infants (at 4 months) show adult-like responses to eye gaze and facial communication cues (Grossmann et al., 2007, 2008) and display similar behavior even when presented with schematic gaze cues (Farroni et al., 2006). For example, in a neuroimaging study with 4-months-old infants, infants' gamma oscillatory activity was different for direct compared to averted gaze, in right frontotemporal regions, similar to adults (Grossmann et al., 2007). Furthermore, mutual gaze and eyebrow raise together with a smile (when mutual gaze was established) elicited similar neural activations, and the eyebrow raise with a smile led to this activation only when it was preceded by mutual gaze, so only when the infant was directly addressed. Possibly, this activation was only elicited when the cue was interpreted as ostensive (and communicative; Grossmann et al., 2008).

Eye gaze may function by facilitating infants' general attention and arousal, thereby increasing their receptivity in social interactions that foster learning. Extensive neuroimaging work has pointed at multiple neural correlates that differ as a function of eye gaze cues and which index attentional processes. For instance, Reid et al. (2004) showed that 4-months-old infants had enhanced positive slow wave (PSW) responses in their ERP signals to objects that were previously not cued with the experimenter's gaze, compared to cued objects. The PSW component is related to memory processes and stimulus encoding and has been found to be larger for novel objects and faces compared to already processed items (de Haan and Nelson, 1999). The authors, thus, argued that the infants needed to perform additional memory updating for the uncued objects, giving rise to the enhanced PSW. This effect was further modulated by the nature of the social cue (Michel et al., 2019) and familiarity of the adult, since in one study with 4-months-old infants, an enhanced PSW was only observed for uncued objects after objects were presented by the caregiver (Hoehl et al., 2012). This finding suggests that cues used by the caregiver might result in enhanced learning, possibly because of an additional increase in processing capacity and/or an increase in attention when interacting with a familiar adult. It should also be noted that older infants might benefit from caregivers' and strangers' eye gaze cues to a similar extent, since it has also been demonstrated that infants between 4 and 6 months show a stranger preference when following gaze (Gredeback et al., 2010).

Further, the negative central (Nc) component, which is taken to reflect attentional arousal and attentional orienting to salient stimuli (Richards et al., 2010), was found to be enhanced in response to objects that were previously cued by the adult's eye gaze and when joint attention was established by mutual gaze before directing gaze to the object. For example, Parise et al. (2008) reported that 5-months-old had significantly larger Nc components in the left frontocentral regions in response to objects that were presented with joint attention (alternating gaze between infant and object after sharing mutual gaze with the infant) compared to the non-joint attention condition (no mutual gaze, looking at object only) during the familiarization phase (Parise et al., 2008). Hoehl et al. (2008b) presented similar results with 3-months-old, showing increased Nc for objects presented with direct gaze and a fearful expression. Findings of Wu et al. (2014) suggest that 8-months-old did learn more about the location of multimodal objects when ostensive cues, such as a video showing a person with direct eye gaze (while also verbally addressing the infant) preceded non-social attentional cues (flashing squares) in the training phase, even when the face did not turn toward the cued location. Similarly, a small sample EEG study suggested that “joint engagement,” which presumably entails more than gaze cues (e.g., gestures and facial expression), led to a larger frontal positive component for objects presented with joint engagement and a larger Nc for objects presented without, indicating more familiarity for objects presented with joint engagement (Hutman et al., 2016). However, establishing a causal role for eye gaze in this observed enhancement effect is not possible in these studies due to coinciding ostensive cues (eye gaze presented together with verbal cues or gestures).

The studies we identified demonstrated that infants show differential brain states during object processing and social interaction with or without joint attention, involving direct eye contact, with an adult (Senju et al., 2006; Hoehl et al., 2014a,b; Michel et al., 2015; Urakawa et al., 2015). For example, Striano et al. (2006b) demonstrated that eye contact established before joint attentional periods during object viewing led to enhanced Nc in 9-months-old, reflecting attentional orienting or attentional arousal. This can then lead to more successful information encoding due to the channeling of limited attentional resources to the relevant aspects of information. Similarly, enhanced Ncs for objects not previously cued with adult's eye gaze shift or head direction were observed in 4-months-old (Hoehl et al., 2014b). Further, 9-months-old infants showed desynchronization of alpha oscillatory activity when viewing objects with an adult, only when the adult engaged in direct eye contact with the infant prior to orienting to the object (Hoehl et al., 2014a), similar to findings of joint attention studies with adults (Lachat et al., 2012). Similar results were observed for 4- and 9-months-old oscillatory activity for object-directed gaze (Michel et al., 2015), which was interpreted as a reflection of infants' developing executive attention control networks. The desynchronization of alpha-band activity, in the context of joint attention, is taken to reflect cortical excitation, attentional suppression of external input in order to focus on relevant information (Ward, 2003), and interestingly, an activation of a generic semantic knowledge system in adults (Klimesch, 2012). Thus, Michel et al. (2015) tentatively concluded that this desynchronization effect could reflect infants' enhanced receptive state of semantic knowledge transmission, which was activated by the use of ostensive gaze cues, thereby offering an interpretation of the attentional arousal effect in terms of natural pedagogy. This proposal requires further investigation.

The difference in neural responses to objects could also arise from infants' differential neural processing of the adult's gaze in relation to the object. Infants processed the experimenter's gaze differentially when her gaze was directed to an object compared to when gaze was averted from an object (Hoehl et al., 2008a). Object-directed gaze led to an enhanced positive slow wave (PSW), while object-averted gaze elicited a more enhanced Nc, with its peak occurring significantly later compared to object-directed gaze (Hoehl et al., 2008a; Wahl et al., 2019). These results suggest that object-directed gaze might be encoded faster and require less attentional resources as reflected by the latency and amplitude of the Nc and may promote better memory encoding as reflected by the enhanced PSW. These could create opportunities for better processing of consequent environmental stimuli. Gaze cues in the context of joint attention (as in Striano et al., 2006b) might also affect the long-term retention of information about objects. Nine-months-old positive components (Pb; positive deflection between 200 and 400 ms, possibly reflecting contextual processing and expectation of an event) differed as a function of whether infants were familiarized with objects in joint attention or non-joint attention contexts. Similar effects were observed in another study both immediately and 1 week after familiarization (Kopp and Lindenberger, 2011), although in this study, two ostensive cues, direct eye contact and infant-directed speech, were conflated in the joint attention context.

##### Eye gaze in infants' learning: special or “just” attention?

Overall, studies testing infants' processing of eye gaze cues, mostly in relation to objects, suggest that eye gaze cues might facilitate learning by enhancing attention and memory encoding. However, while these studies provide a basis for interpreting eye gaze as a highly salient and advantageous social cue in infants' social communication and learning, they cannot provide a concrete answer to whether such ostensive cues have a special state for infants (i.e., whether they convey meaning over and above other attentional cues). This is because these studies do not directly compare eye gaze to other non-ostensive attentional cues. Our search procedure identified only a small number of studies that directly investigated whether it is the enhanced processing elicited by the social nature of such cues or their (low-level) attention-grabbing features, such as movement, that contribute to learning (Farroni et al., 2000). The results of these studies are, overall, inconsistent. Some report results that support the natural pedagogy theory (i.e., that there is enhanced processing associated with the ostensive nature of eye gaze cues), but others conclude that eye gaze is not more facilitative than other low-level attentional cues.

In support of the natural pedagogy theory, Senju and Csibra (2008) reported differences in how infants responded to the ostensive and non-ostensive cues that preceded an adult's head-turn/gaze switch toward an object. They demonstrated that 6-months-old infants followed an adult's gaze when the gaze switch was preceded by an ostensive cue, such as direct eye contact or infant-directed speech. The 6-months-old infants, however, did not reliably follow gaze when the gaze switch followed a non-ostensive, attention-grabbing cue. Similarly, 8-months-old infants performed more successfully in learning the location of cues in multimodal events when ostensive cues (a face addressing the infants with direct eye contact, accompanied by infant-directed speech) preceded non-social attentional cues (flashing squares). This was true even when the ostensive cue itself did not orient toward the cued location. These results suggest that the ostensive cue helped infants learn from other non-ostensive cues (Wu et al., 2014).

There is also evidence that, during object processing, 4-months-old infants showed sensitivity to eye gaze cues but not to non-social attentional cues, as shown by their enhanced positive slow wave ERP responses to uncued objects (Michel et al., 2019). Further evidence is provided by Parise and Csibra (2013) who illustrated that 5-months-old infants' had overlapping electrophysiological responses to infant-directed speech and direct eye gaze in (pre)frontal regions, similar to adults. As direct eye gaze and infant-directed speech occur in different modalities, they do not have any common low-level physical properties; the overlapping brain activity must thus be due to another mechanism than the perception of low-level stimulus features. The authors hypothesized that if the observed activity in these regions was driven by increased attention, the combination of the two signals should produce a greater activity; however, the two signals gave rise to the same activity, with an early latency, as either signal in isolation. The authors took this obligatory response with an early latency as indicating infants' “fast and rudimentary interpretation” of the stimuli as ostensive, rather than resulting from the stimuli's low-level attention-grabbing features (Parise and Csibra, 2013). Interestingly, the combination of one ostensive and one non-ostensive signal, such as infant-directed speech (IDS) and averted gaze, did not cancel out the effects, but this might be due to the fact that the infants were too young to inhibit the early response to one ostensive signal, even if the accompanying cue in the other modality did not corroborate its ostensive nature.

Consistent with these results, 4-months-old infants' object processing was influenced by social cues (Wahl et al., 2013). Here, the effects were compared of directionally cueing objects with either an inanimate object (e.g., a car) or a human face. When the human face provided the cues, infants showed increased attention to, and processing of, uncued objects compared to the cued ones., This was indicated by increased looking times and enhanced Nc amplitudes for the uncued object, suggesting that the cued object was processed more efficiently (Wahl et al., 2013). When the cues were provided by the car, there were no looking time differences and only marginally significant ERP effects. However, in a later study, the authors raised concerns about the perceptual similarities between cars and human faces (features of the car stimuli that could be interpreted as face-like features by infants, such as side mirrors) and infants' possible familiarity with cars. Instead, they used a box with either a checkerboard pattern or with eyes as the central cue (Michel et al., 2019). Their results revealed a more robust enhanced PSW in response to uncued objects when cued with eyes (although the Nc component did not differ between conditions). This suggests that social cues (even schematic patterns thereof) might play a specific role in infants' learning about objects, over and above other attention-grabbing, dynamic cues. Interestingly, another study found increased looking times and an enhanced Nc in response to objects that were previously not cued by isolated eyes gazing at the object (without a face). An enhanced slow-wave positivity was found in response to the object-directed (vs. averted) isolated eyes cue, suggesting that eyes only might be sufficient to facilitate object encoding (Wahl et al., 2019). This might also depend on the contrast polarity of the schematic images (black circles on white background vs. white circles on black background; Michel et al., 2017 also see Jessen and Grossmann, 2014).

The studies summarized above have been interpreted as support for natural pedagogy theory by many, since they seem to show differential (and sometimes enhanced) reactions or learning in the presence of ostensive cues. These reactions or learning, accordingly, do not result from the presence of low-level attentional, non-ostensive cues only (Csibra and Gergely, 2011). However, there are also studies that report no difference in differential gaze following or learning preceded by ostensive and non-ostensive conditions. These usually conclude that eye gaze is simply an attentional cue, which is highly salient for the infants from an early age on as evident by their automatic-like orientation toward its direction. On this view, eye gaze acts as a powerful attention modulator because it highlights to the infant where to attend in the noisy environment and which relevant information pieces are available in the environment to learn. On this view, eye contact may enhance infants' overall social attention to the environment and communication partner. This facilitates learning (Szufnarowska et al., 2014), but it does not necessarily hold a unique (ostensive) meaning for the infant to the extent that they treat it differently from other non-social attentional cues. Moreover, gaze following does not necessarily signal that infants understand the interlocutor's communicative intent.

Our review identified a number of studies (*n* = 5) showing that ostensive cues (such as eye contact but also infant-directed speech) do not necessarily need to be present for infants to follow an adult's gaze to a particular part of the environment. For instance, de Bordes et al. (2013) showed that 20-months-old infants followed the adult's gaze equally well after eye contact was established as when adult's eyes were made salient by placing colorful moving dots over them but no direct eye contact was present (although note that the adult's gaze was still directed at the infant, even when it was covered by the blinking dots, so infants might still have interpreted this condition as direct eye contact). In addition, these infants were substantially older, and thus capable of more sophisticated gaze cuing, than the children in many other studies). Similarly, it has been suggested that 6-months-old infants follow gaze in different ostensive and non-ostensive contexts, when the adult's action preceding the gaze orienting head turn was attention grabbing for the infant, irrespective of whether this action was ostensive or not (Szufnarowska et al., 2014; Gredebäeck et al., 2018). Moreover, recent evidence has shown that infants between 11 and 24 months and their parents can coordinate visual attention without gaze following, by relying on the coordination of eyes and hands in naturalistic, complex settings (Yu and Smith, 2017). These results suggest that it might be domain-general attention-based mechanisms, rather than the special status of the eyes, that explains why infants follow, and learn from, adults' gaze. Such domain-general accounts are also used to explain infants' ability at 9 months to learn from non-social cues, such as shapes, as well as from social cues when learning about object statistics (Barry et al., 2015). Other related theories have also proposed that infants acquire sensitivity to eye gaze through reinforcement learning by 9 months, without assigning a privileged status to eye gaze (Moore et al., 1997). Finally, one study found that 9-months-old infants learned object sequences equally well from social and non-social cues (Barry et al., 2015).

In sum, taken together, there exists a considerable body of literature suggesting that gaze is a highly attention-grabbing cue, to which infants show early sensitivity. The literature reviewed in the first part of this section presents quite convincing indirect evidence for differential processing and learning as a result of eye gaze cues compared to non-social attentional cues. However, the studies presented in the second half, which directly compared infants' tendency to follow the adult's gaze in ostensive and non-ostensive conditions, provided mixed evidence about the question of whether eye gaze is more than simply a high attention-grabbing cue. Thus, we only tentatively conclude that learning is especially enhanced when infants are addressed by ostensive signals, which may support the hypothesis that gaze cues facilitate infants' attention, arousal, and memory mechanisms in a way that other attentional cues do not.

## General Discussion

### Summary of Results

Our review identified studies assessing the role of eye gaze in infants' language learning in four different domains: (1) vocabulary development, (2) word–object mappings, (3) object processing, and (4) speech processing. We then discussed the mechanisms by which eye gaze might play a role in infants' learning in these domains. With regards to vocabulary development, it appears that there is a strong association between infants' pre-linguistic communicative skills, such as following an adult's gaze direction, and their later receptive and expressive vocabulary. We suggest that this could be a cumulative result of enhanced processing due to eye gaze in the other domains we discussed, namely, word–object mappings, object processing, and speech processing. Eye gaze seems to facilitate the formation and retention of word-object mappings, as shown by both behavioral and neuroimaging studies, although the presence of other social cues coinciding with eye gaze in many of the studies makes it difficult to interpret whether the facilitation is indeed due to eye gaze. Similarly, object processing was found to be enhanced by eye gaze cues, although caution must be applied as, here too, some studies did not manipulate eye gaze in isolation. Finally, the limited evidence with regards to infants' speech processing suggests that infants might process speech sounds differently when accompanied by ostensive cues, such as eye gaze and that infants who shifted gaze learned more from the speech stream, as indexed by their phonemic discrimination. In general, therefore, it seems that eye gaze can act as a powerful social cue in guiding infants' learning in different cognitive domains that are linked to language development.

There are two types of eye gaze cues in communication. The first is gaze alternation (the speaker alternates their gaze between the listener and the object being referred to), which invites the social partner to gaze follow. The second is simply establishing mutual eye contact with the partner. Many of the studies identified by our review focused on the first of these—gaze following. Such studies treat eye gaze as a spatiotemporal referential cue that signals the listener where to attend in the environment, so that referent–label associations may be formed. In that sense, the observed effects may be limited to certain domains or tasks that require a spatial referent in the environment, such as word–object mapping. However, it is also possible that the observed facilitatory effects of eye gaze reflect a general learning enhancement mechanism that is not confined to spatiotemporal mapping driven by gaze following but might follow the establishment of mutual eye contact. Our review provided support for a general enhancement mechanism. First, eye gaze in the form of gaze following seems to have a facilitatory role only when it is preceded by mutual eye contact. For instance, infants processed objects differently in live joint attentional contexts with an adult as a function of whether the adult provided eye contact or not, such that they had greater attentional mechanisms involved in object processing when the adult offered eye contact (Parise et al., 2008; Hoehl et al., 2014a). Moreover, the facilitatory effects are observed in domains that do not necessarily require spatial cues in the environment, such as speech processing, which was enhanced when the adult spoke to the infant in an infant-directed manner while providing mutual eye contact (Parise et al., 2011). Taken together, we suggest that eye gaze may have a general learning enhancing function in infants' (language) learning, such that the enhanced attentional and arousal mechanisms are observed when gaze following is accompanied by eye contact (which happens most frequently in natural interactions), and across domains. However, as few studies investigated the effects of eye gaze in domains other than object processing and word–object associations, the evidence to support our interpretation is limited.

Here, a related point that arose from our review is whether eye gaze holds a special status in infants' learning as a highly specialized socio-cognitive cue that is different from other attentional cues. Only a few studies directly assessed this question. However, once again, taking the evidence in all domains into consideration, the balance of evidence suggests that eye gaze may be a special attentional cue in that it facilitates learning to an extent that other low-level attentional cues cannot. Yet, we would argue, a developmental approach is necessary to fully understand the mechanisms by which children use eye gaze in learning. Our reading of the evidence to date is that children do not start out by treating eye gaze as an ostensive and referential cue but gradually learn to treat it as such throughout the first 2 years of life. Young infants have a preference for direct gaze, and for upright faces (Farroni et al., 2002), show early specialization of cortical regions involved in the processing of gaze cues and show mature neural responses to such cues. However, this does not mean that eye gaze has a special status in human ontogeny from the start. Eye gaze could act as an attention-grabbing, albeit highly salient, cue early in development but not yet be treated as ostensive or referential. The development of an ostensive, referential understanding of eye gaze, instead, seems to develop between 9 and 12 months, as demonstrated by studies showing that infants follow gaze in conditions that signal referential, object-directed information by this age (Butler et al., 2000; Brooks and Meltzoff, 2002; Caron et al., 2002; Woodward, 2003; D'Entremont and Morgan, 2006; Johnson et al., 2007), and by neuroimaging studies showing that infants process referential information in an adult-like way by 9 months (Senju et al., 2006). Their ability to use eye gaze for object labeling, in an adultlike way, however, seems to come even later, at about 24 months of age.

This interpretation is also consistent with research showing that 8-months-old infants learned from social cues, whereas 4-months-old learned from non-social attention-grabbing cues, suggesting that “learning to learn” from social cues might be a skill that develops during infancy (Wu and Kirkham, 2010), and their sensitivity to social cues may develop gradually through the development of attention control, memory, and information processing networks (Yurovsky and Frank, 2017). Note, though, that a developmental explanation would not necessarily predict a linear developmental pattern. Multiple factors are likely to contribute to when and how infants make use of information provided by eye gaze cues, including the nature of the task and/or interaction they are engaged in. For example, it may be that older infants do not need to use eye gaze cues to solve simple object processing tasks (Striano et al., 2006a) but might still benefit from them in more complex settings, such as naturalistic interaction.

In fact, eye gaze as a social cue, in the form of mutual eye contact or gaze direction, rarely occurs in isolation in natural social–communicative contexts. Infant–adult social interactions are rich in a number of social signals that help infants in learning from others, and eye gaze often co-occurs with other ostensive cues, such as infant-directed speech and pointing. While this poses a problem for studying the role of eye gaze in isolation in infant learning, it also provides an important area for further research: to identify which kinds of rich communicative settings are optimal for learning. If we also consider recent evidence showing that infants show enhanced sustained attentional states during joint attention episodes (Yu et al., 2019), it will also be important to consider the role of the infants' endogenous attentional states within the context of parent–infant interactions.

In sum, eye gaze, both in the form of eye contact and gaze following, may direct and help infants in sustaining their attention and thus learn about relevant information in the environment. The involvement of attentional processes further corroborates the possibility that the use of eye gaze cues might serve infants' learning by highlighting the information to be attended and channeling their attentional resources. Further, eye gaze may, over the course of the first 2 years of life, develop into a truly ostensive, referential cue that enhances language learning across the board. However, further work is needed to fully understand the mechanisms behind the observed effects.

### Limitations

In this review, we assessed the available experimental evidence on the effects of eye gaze on infants' learning and attention. Therefore, a number of other domains, such as face processing and emotion understanding were excluded from our analyses. Although this was intentionally done, it is important to acknowledge that infants' emerging social skills, such as face processing and understanding of others' emotions and intentions have effects on their cognitive abilities and language development, such as theory of mind development and mental state vocabulary. Separate reviews of these literature may throw additional light onto some of the issues discussed here.

Furthermore, as our review focuses on eye gaze, we narrowed our key search terms to include eye gaze or eye contact, rather than searching for literature on joint attention and learning. This allows us to focus on the role of gaze as intended but means we exclude literature using composite scores that include gaze as one of the components [e.g., studies that use the Early Social Communication Scales (ESCS) composite scores rather the result for the individual gaze questions]. A future review, building on the present one, and collating information from a range of joint attentional tasks, would be a useful addition to the literature.

Another point is to note that the age at which infants were tested differed substantially across different learning domains. For example, in most of the studies on word–referent mapping (Word–Object Mapping), infants are older than in the studies on object processing (Object Processing). This is not unexpected, as children are rarely tested on their abilities to form word–object associations before 12 months and have difficulties to form these associations before 13 months (Woodward et al., 1994; Werker et al., 1998). However, the developmental differences make it difficult to compare the evidence across different domains, thus limiting our ability to draw concrete conclusions about the timescale of development.

Another important limitation is that some of the findings we report have not yet been replicated. Relatedly, we also observed that, for many studies, attrition posed a major challenge to the interpretation of the findings. For instance, Parise et al. (2008) tested 69 5-month-olds but were able to include data from only 15 infants. Fifty-two of those infants were excluded due to fussiness or for failing to reach a certain threshold that allowed for an adequate averaging of the ERP data. While the authors acknowledged this high dropout rate and argued that it was due to the relatively high task demands of their study, such high dropout rates are not unusual in the reviewed literature. This is a concern for the neuroimaging evidence in particular (e.g., Senju et al., 2006, who retained only 10/33 infants tested in the final dataset) but also for the behavioral studies. For behavioral studies, the dropout rates seem to differ with the numbers of participants recruited for the study and the task requirements (e.g., compare Cleveland et al., 2007 who retained 16/22 of the infants tested, to Gredebäeck et al., 2018 where 94/95 of the infants were included in the final dataset). Thus, there are questions of generalizability to be answered; for instance, is this evidence reflective only of a selected group of infants who seem to have better attentional spans as well as possibly better perceptual capacities?

### Future Directions

Some additional themes that emerged from our search were not discussed in depth above because the literature was too sparse to draw reliable conclusions. For instance, in Speech Processing, we discussed the literature on speech processing, but the section is small because the literature is thin, leaving many unanswered questions. We need more of such work, which has the potential to address, directly, the question of whether gaze cues yield a general processing enhancement effect.

Another issue concerns the effect of live social interaction on language learning. Kuhl (2007) has suggested that social interaction is crucial for language learning, such that infants only learn to discriminate non-native phonemes in the context of live communication, not from videotaped interactions. This could be due to increased attention and arousal during live interactions compared to videotaped tutoring or to live situations being richer in social referential cues (such as eye gaze) that promote learning (although note that these two explanations are not mutually exclusive, since social cues might lead to enhanced attention). While many of the studies reported here tested language learning from audio or audiovisual stimuli presented in laboratory settings, it is possible that learning more complex linguistic information requires the presence of a live speaker who can convey the referential nature of the communication.

It is not currently possible to systematically compare the results of the studies that had a live interaction paradigm to those using prerecorded stimuli, since such studies had many methodological differences other than these variables, such as age and number of infants tested. Further research is needed to compare live interaction and classical lab studies that can assess the importance of natural interaction in different aspects of language learning. There has recently been a move toward studying social interaction in a more ecologically valid context and to consider how interpersonal communication affects information transfer, taking bidirectional influences between the partners into account. Recent dual-imaging work showed that eye gaze enhanced interpersonal brain synchrony between adult–infant pairs, in both live interaction and in a prerecorded condition (Leong et al., 2017). This provides possible explanations of mechanisms of how gaze functions to create learning opportunities for young infants during social interactions, perhaps by facilitating interpersonal synchrony through phase-resetting oscillatory activity and thereby putting children in a receptive state. Studying interpersonal neural dynamics in the context of infant learning is a fruitful area for further work.

Finally, to fully understand the socio-cognitive mechanisms that underlie the effects of eye gaze, we need more work directly testing whether eye gaze is interpreted as special, and ostensive, by infants or is treated as simply another attentional cue. Such studies must take account of the fact that there may be developmental- and task-specific differences in how children react to eye gaze cues. It is probable that eye gaze serves different purposes at different ages for infants, starting as a salient attentional cue, and perhaps gaining a special status as infants develop. Further developmental work is required to establish the viability of these hypotheses.

## Data Availability Statement

The original contributions presented in the study are included in the article/Supplementary Material, further inquiries can be directed to the corresponding author/s.

## Author Contributions

MÇ, TS, and CR contributed to the conception and design of the study. MÇ was primarily responsible for the literature search, the study selection process, data extraction, and wrote the first draft of the manuscript. TS and CR supervised the study. All authors contributed to manuscript revision and read and approved the submitted version.

## Conflict of Interest

The authors declare that the research was conducted in the absence of any commercial or financial relationships that could be construed as a potential conflict of interest.

## References

[B1] Akhtar N. Gernsbacher M. A. (2007). Joint attention and vocabulary development: a critical look. Lang. Linguist. Compass 1, 195–207. 10.1111/j.1749-818X.2007.00014.x 25505491 PMC4258841

[B2] Barry R. A. Estes K. G. Rivera S. M. (2015). Domain general learning: infants use social and non-social cues when learning object statistics. Front. Psychol. 6:551. 10.3389/fpsyg.2015.00551 25999879 PMC4420800

[B3] Barry-Anwar R. A. Burris J. L. Estes K. G. Rivera S. M. (2017). Caregivers and strangers: the influence of familiarity on gaze following and learning. Infant Behav. Dev. 46, 46–58. 10.1016/j.infbeh.2016.11.005 27898343

[B4] Batki A. Baron-Cohen S. Wheelwright S. Connellan J. Ahluwalia J. (2000). Is there an innate gaze module? Evidence from human neonates. Infant Behav. Dev. 23, 223–229. 10.1016/S0163-6383(01)00037-6

[B5] Beuker K. T. Rommelse N. N. J. Donders R. Buitelaar J. K. (2013). Development of early communication skills in the first two years of life. Infant Behav. Dev. 36, 71–83. 10.1016/j.infbeh.2012.11.001 23261791

[B6] Brooks R. Meltzoff A. N. (2002). The importance of eyes: how infants interpret adult looking behavior. Dev. Psychol. 38, 958–966. 10.1037/0012-1649.38.6.958 12428707 PMC1351351

[B7] Brooks R. Meltzoff A. N. (2005). The development of gaze following and its relation to language. Dev. Sci. 8, 535–543. 10.1111/j.1467-7687.2005.00445.x 16246245 PMC3640988

[B8] Brooks R. Meltzoff A. N. (2008). Infant gaze following and pointing predict accelerated vocabulary growth through two years of age: a longitudinal, growth curve modeling study. J. Child Lang. 35, 207–220. 10.1017/S030500090700829X 18300435

[B9] Brooks R. Meltzoff A. N. (2015). Connecting the dots from infancy to childhood: a longitudinal study connecting gaze following, language, and explicit theory of mind. J. Exp. Child Psychol. 130, 67–78. 10.1016/j.jecp.2014.09.010 25462032 PMC7089676

[B10] Butler S. C. Caron A. J. Brooks R. (2000). Infant understanding of the referential nature of looking. J. Cogn. Dev. 1, 359–377. 10.1207/S15327647JCD0104_01

[B11] Caron A. J. Butler S. Brooks R. (2002). Gaze following at 12 and 14 months: do the eyes matter? Br. J. Dev. Psychol. 20, 225–239. 10.1348/026151002166424

[B12] Carpenter M. Nagell K. Tomasello M. (1998). Social cognition, joint attention, and communicative competence from 9 to 15 months of age. Monogr. Soc. Res. Child Dev. 63:i–vi, 1–143. 10.2307/1166214 9835078

[B13] Charman T. Baron-Cohen S. Swettenham J. Baird G. Cox A. Drew A. (2000). Testing joint attention, imitation, and play as infancy precursors to language and theory of mind. Cogn. Dev. 15, 481–498. 10.1016/S0885-2014(01)00037-5

[B14] Cleveland A. Schug M. Striano T. (2007). Joint attention and object learning in 5-and 7-month-old infants. Infant Child Dev. 16, 295–306. 10.1002/icd.508

[B15] Cleveland A. Striano T. (2007). The effects of joint attention on object processing in 4- and 9-month-old infants. Infant Behav. Dev. 30, 499–504. 10.1016/j.infbeh.2006.10.009 17610957

[B16] Conboy B. T. Brooks R. Meltzoff A. N. Kuhl P. K. (2015). Social interaction in infants' learning of second-language phonetics: an exploration of brain-behavior relations. Dev. Neuropsychol. 40, 216–229. 10.1080/87565641.2015.1014487 26179488 PMC4824050

[B17] Csibra G. (2010). Recognizing communicative intentions in infancy. Mind Lang. 25, 141–168. 10.1111/j.1468-0017.2009.01384.x

[B18] Csibra G. Gergely G. (2009). Natural pedagogy. Trends Cogn. Sci. 13, 148–153. 10.1016/j.tics.2009.01.005 19285912

[B19] Csibra G. Gergely G. (2011). Natural pedagogy as evolutionary adaptation. Philos. Trans. R. Soc. Lond. B Biol. Sci. 366, 1149–1157. 10.1098/rstb.2010.0319 21357237 PMC3049090

[B20] Csibra G. Volein A. (2008). Infants can infer the presence of hidden objects from referential gaze information. Br. J. Dev. Psychol. 26, 1–11. 10.1348/026151007X185987

[B21] de Bordes P. F. Cox R. F. A. Hasselman F. Cillessen A. H. N. (2013). Toddlers' gaze following through attention modulation: intention is in the eye of the beholder. J. Exp. Child Psychol. 116, 443–452. 10.1016/j.jecp.2012.09.008 23073365

[B22] de Haan M. Nelson C. A. (1999). Brain activity differentiates face and object processing in 6-month-old infants. Dev. Psychol. 35, 1113–1121. 10.1037/0012-1649.35.4.1113 10442879

[B23] De Schuymer L. De Groote I. Striano T. Stahl D. Roeyers H. (2011). Dyadic and triadic skills in preterm and full term infants: a longitudinal study in the first year. Infant Behav. Dev. 34, 179–188. 10.1016/j.infbeh.2010.12.007 21185604

[B24] D'Entremont B. Hains S. M. J. Muir D. W. (1997). A demonstration of gaze following in 3- to 6-month-olds. Infant Behav. Dev. 20, 569–572. 10.1016/S0163-6383(97)90048-5

[B25] D'Entremont B. Morgan R. (2006). Experience with visual barriers and its effects on subsequent gaze-following in 12- to 13-month-olds. Br. J. Dev. Psychol. 24, 465–475. 10.1348/026151005X51248

[B26] D'Odorico L. Cassibba R. Salerni N. (1997). Temporal relationships between gaze and vocal behavior in prelinguistic and linguistic communication. J. Psycholinguist. Res. 26, 539–556. 10.1023/A:1025027830861 9329205

[B27] Donnellan E. Bannard C. McGillion M. L. Slocombe K. E. Matthews D. (2020). Infants' intentionally communicative vocalizations elicit responses from caregivers and are the best predictors of the transition to language: a longitudinal investigation of infants' vocalizations, gestures and word production. Dev. Sci. 23:e12843. 10.1111/desc.12843 31045301

[B28] Farroni T. Csibra G. Simion G. Johnson M. H. (2002). Eye contact detection in humans from birth. Proc. Natl. Acad. Sci. U.S.A. 99, 9602–9605. 10.1073/pnas.152159999 12082186 PMC123187

[B29] Farroni T. Johnson M. H. Brockbank M. Simion F. (2000). Infants' use of gaze direction to cue attention: the importance of perceived motion. Vis. Cogn. 7, 705–718. 10.1080/13506280050144399

[B30] Farroni T. Johnson M. H. Csibra G. (2004). Mechanisms of eye gaze perception during infancy. J. Cogn. Neurosci. 16, 1320–1326. 10.1162/0898929042304787 15509381

[B31] Farroni T. Menon E. Johnson M. H. (2006). Factors influencing newborns' preference for faces with eye contact. J. Exp. Child Psychol. 95, 298–308. 10.1016/j.jecp.2006.08.001 17030037

[B32] Fenson L. Marchman V. A. Thal D. J. Dale P. S. Reznick J. S. Bates E. (2007). MacArthur-Bates Communicative Development Inventories: User's Guide and Technical Manual, 2nd Edn. Oxford: Oxford Brookes University.

[B33] Gogate L. J. Bolzani L. H. Betancourt E. A. (2006). Attention to maternal multimodal naming by 6- to 8-month-old infants and learning of word-object relations. Infancy 9, 259–288. 10.1207/s15327078in0903_1 33412680

[B34] Graham S. A. Nilsen E. S. Collins S. Olineck K. (2010). The role of gaze direction and mutual exclusivity in guiding 24-month-olds' word mappings. Br. J. Dev. Psychol. 28, 449–465. 10.1348/026151009X424565 20481397

[B35] Graham S. A. Nilsen E. S. Friesen C. K. Johnson J. (2011). Examining the role of attention and intention in two-year-olds' acquisition of novel words. Enfance 63, 311–328. 10.4074/S0013754511003041

[B36] Gredeback G. Fikke L. Melinder A. (2010). The development of joint visual attention: a longitudinal study of gaze following during interactions with mothers and strangers. Dev. Sci. 13, 839–848. 10.1111/j.1467-7687.2009.00945.x 20977555

[B37] Gredebäeck G. Astor K. Fawcett C. (2018). Gaze following is not dependent on ostensive cues: a critical test of natural pedagogy. Child Dev. 89, 2091–2098. 10.1111/cdev.13026 29315501

[B38] Grossmann T. Johnson M. H. Farroni T. Csibra G. (2007). Social perception in the infant brain: gamma oscillatory activity in response to eye gaze. Soc. Cogn. Affect. Neurosci. 2, 284–291. 10.1093/scan/nsm025 18985134 PMC2566755

[B39] Grossmann T. Johnson M. H. Lloyd-Fox S. Blasi A. Deligianni F. Elwell C.. (2008). Early cortical specialization for face-to-face communication in human infants. Proc. Biol. Sci. 275, 2803–2811. 10.1098/rspb.2008.0986 18755668 PMC2572680

[B40] Hirotani M. Stets M. Striano T. Friederici A. D. (2009). Joint attention helps infants learn new words: event-related potential evidence. Neuroreport 20, 600–605. 10.1097/WNR.0b013e32832a0a7c 19287321

[B41] Hoehl S. Michel C. Reid V. M. Parise E. Striano T. (2014a). Eye contact during live social interaction modulates infants' oscillatory brain activity. Soc. Neurosci. 9, 300–308. 10.1080/17470919.2014.884982 24506533

[B42] Hoehl S. Reid V. Mooney J. Striano T. (2008a). What are you looking at? Infants' neural processing of an adult's object-directed eye gaze. Dev. Sci. 11, 10–16. 10.1111/j.1467-7687.2007.00643.x 18171361

[B43] Hoehl S. Wahl S. Michel C. Striano T. (2012). Effects of eye gaze cues provided by the caregiver compared to a stranger on infants' object processing. Dev. Cogn. Neurosci. 2, 81–89. 10.1016/j.dcn.2011.07.015 22682729 PMC6987711

[B44] Hoehl S. Wahl S. Pauen S. (2014b). Disentangling the effects of an adult model's eye gaze and head orientation on young infants' processing of a previously attended object. Infancy 19, 53–64. 10.1111/infa.12035

[B45] Hoehl S. Wiese L. Striano T. (2008b). Young infants' neural processing of objects is affected by eye gaze direction and emotional expression. PLoS ONE 3:e2389. 10.1371/journal.pone.0002389 18545689 PMC2405932

[B46] Hollich G. J. Hirsh-Pasek K. Golinkoff R. M. Brand R. J. Brown E. Chung H. L.. (2000). Breaking the language barrier: an emergentist coalition model for the origins of word learning. Monogr. Soc. Res. Child Dev. 65, i–vi, 1–135. 12467096

[B47] Hood B. M. Willen J. D. Driver J. (1998). Adult's eyes trigger shifts of visual attention in human infants. Psychol. Sci. 9, 131–134. 10.1111/1467-9280.00024

[B48] Houston-Price C. Plunkett K. Duffy H. (2006). The use of social and salience cues in early word learning. J. Exp. Child Psychol. 95, 27–55. 10.1016/j.jecp.2006.03.006 16677668

[B49] Hutman T. Harrop C. Baker E. Elder L. Abood K. Soares A.. (2016). Joint engagement modulates object discrimination in toddlers: a pilot electrophysiological investigation. Soc. Neurosci. 11, 525–530. 10.1080/17470919.2015.1114966 26527311 PMC4879106

[B50] Jessen S. Grossmann T. (2014). Unconscious discrimination of social cues from eye whites in infants. Proc. Nat. Acad. Sci. U.S.A. 111, 16208–16213. 10.1073/pnas.1411333111 25349392 PMC4234573

[B51] Johnson S. C. Ok S.-J. Luo Y. (2007). The attribution of attention: 9-month-olds' interpretation of gaze as goal-directed action. Dev. Sci. 10, 530–537. 10.1111/j.1467-7687.2007.00606.x 17683339

[B52] Kleinke C. L. (1986). Gaze and eye contact. A research review. Psychol. Bull. 100, 78–100. 10.1037/0033-2909.100.1.78 3526377

[B53] Klimesch W. (2012). α-band oscillations, attention, and controlled access to stored information. Trends Cogn. Sci. 16, 606–617. 10.1016/j.tics.2012.10.007 23141428 PMC3507158

[B54] Kopp F. Lindenberger U. (2011). Effects of joint attention on long-term memory in 9-month-old infants: an event-related potentials study. Dev. Sci. 14, 660–672. 10.1111/j.1467-7687.2010.01010.x 21676087

[B55] Kopp F. Lindenberger U. (2012). Social cues at encoding affect memory in 4-month-old infants. Soc. Neurosci. 7, 458–472. 10.1080/17470919.2011.631289 22047172

[B56] Kuhl P. K. (2007). Is speech learning gated by the social brain? Dev. Sci. 10, 110–120. 10.1111/j.1467-7687.2007.00572.x 17181708

[B57] Lachat F. Hugeville L. Lemarechal J.-D. Conty L. George N. (2012). Oscillatory brain correlates of live joint attention: a dual-EEG study. Front. Hum. Neurosci. 6:156. 10.3389/fnhum.2012.00156 22675297 PMC3365444

[B58] Leong V. Byrne E. Clackson K. Georgieva S. Lam S. Wass S. (2017). Speaker gaze increases information coupling between infant and adult brains. Proc. Natl. Acad. Sci.U.S.A. 114, 13290–13295. 10.1073/pnas.1702493114 29183980 PMC5740679

[B59] Lewkowicz D. J. Hansen-Tift A. M. (2012). Infants deploy selective attention to the mouth of a talking face when learning speech. Proc. Natl. Acad. Sci. U.S.A. 109, 1431–1436. 10.1073/pnas.1114783109 22307596 PMC3277111

[B60] Lloyd-Fox S. Széplaki-Köllod B. Yin J. Csibra G. (2015). Are you talking to me? Neural activations in 6-month-old infants in response to being addressed during natural interactions. Cortex 70, 35–48. 10.1016/j.cortex.2015.02.005 25891796 PMC4636047

[B61] Markus J. Mundy P. Morales M. Delgado C. E. F. Yale M. (2000). Individual differences in infant skills as predictors of child-caregiver joint attention and language. Soc. Dev. 9, 302–315. 10.1111/1467-9507.00127

[B62] Matatyaho D. J. Gogate L. J. (2008). Type of maternal object motion during synchronous naming predicts preverbal infants' learning of word-object relations. Infancy 13, 172–184. 10.1080/15250000701795655 33412725

[B63] McGillion M. Herbert J. S. Pine J. Vihman M. dePaolis R. Keren-Portnoy T.. (2017). What paves the way to conventional language? The predictive value of babble, pointing, and socioeconomic status. Child Dev. 88, 156–166. 10.1111/cdev.12671 27859008

[B64] Michel C. Pauen S. Hoehl S. (2017). Schematic eye-gaze cues influence infants' object encoding dependent on their contrast polarity. Sci. Rep. 7:7347. 10.1038/s41598-017-07445-9 28779121 PMC5544696

[B65] Michel C. Stets M. Parise E. Reid V. M. Striano T. Hoehl S. (2015). Theta- and alpha-band EEG activity in response to eye gaze cues in early infancy. Neuroimage 118, 576–583. 10.1016/j.neuroimage.2015.06.042 26095092

[B66] Michel C. Wronski C. Pauen S. Daum M. M. Hoehl S. (2019). Infants' object processing is guided specifically by social cues. Neuropsychologia 126, 54–61. 10.1016/j.neuropsychologia.2017.05.022 28551464

[B67] Moher D. Liberati A. Tetzlaff J. Altman D. G. PRISMA Group. (2009). Preferred reporting items for systematic reviews and meta-analyses: the PRISMA statement. PLoS Med. 6:e1000097 10.1371/journal.pmed.1000097 19621072 PMC2707599

[B68] Moll H. Tomasello M. (2004). 12- and 18-month-old infants follow gaze to spaces behind barriers. Dev. Sci. 7, F1–F9. 10.1111/j.1467-7687.2004.00315.x 15323111

[B69] Moore C. Angelopoulos M. Bennett P. (1997). The role of movement in the development of joint visual attention. Infant Behav. Dev. 20, 83–92. 10.1016/S0163-6383(97)90063-1

[B70] Moore C. Angelopoulos M. Bennett P. (1999). Word learning in the context of referential and salience cues. Dev. Psychol. 35, 60–68. 10.1037/0012-1649.35.1.60 9923464

[B71] Morales M. Mundy P. Delgado C. E. F. Yale M. Messinger D. Neal R. (2000a). Responding to joint attention across the 6-through 24-month age period and early language acquisition. J. Appl. Dev. Psychol. 21, 283–298. 10.1016/S0193-3973(99)00040-4

[B72] Morales M. Mundy P. Delgado C. E. F. Yale M. Neal R. Schwartz H. K. (2000b). Gaze following, temperament, and language development in 6-month-olds: a replication and extension. Infant Behav. Dev. 23, 231–236. 10.1016/S0163-6383(01)00038-8

[B73] Morales M. Mundy P. Rojas J. (1998). Following the direction of gaze and language development in 6-month-olds. Infant Behav. Dev. 21, 373–377. 10.1016/S0163-6383(98)90014-5

[B74] Mundy P. Block J. Delgado C. Pomares Y. Van Hecke A. V. Parlade M. V. (2007). Individual differences and the development of joint attention in infancy. Child Dev. 78, 938–954. 10.1111/j.1467-8624.2007.01042.x 17517014 PMC2654237

[B75] Mundy P. Delgado C. Goldstein J. Parlade M. Hogan A. Seibert J. (2003a). Early Social Communication Scales (ESCS). Coral Gables, FL: University of Miami.

[B76] Mundy P. Fox N. Card J. (2003b). EEG coherence, joint attention and language development in the second year. Dev. Sci. 6, 48–54. 10.1111/1467-7687.00253

[B77] Mundy P. Gomes A. (1998). Individual differences in joint attention skill development in the second year. Infant Behav. Dev. 21, 469–482. 10.1016/S0163-6383(98)90020-0

[B78] Niedzwiecka A. Ramotowska S. Tomalski P. (2018). Mutual gaze during early mother-infant interactions promotes attention control development. Child Dev. 89, 2230–2244. 10.1111/cdev.12830 28510324

[B79] O'Connell L. Poulin-Dubois D. Demke T. Guay A. (2009). Can infants use a nonhuman agent's gaze direction to establish word-object relations? Infancy 14, 414–438. 10.1080/15250000902994073 32693449

[B80] Okumura Y. Kanakogi Y. Kanda T. Ishiguro H. Itakura S. (2013a). The power of human gaze on infant learning. Cognition 128, 127–133. 10.1016/j.cognition.2013.03.011 23672983

[B81] Okumura Y. Kanakogi Y. Kanda T. Ishiguro H. Itakura S. (2013b). Infants understand the referential nature of human gaze but not robot gaze. J. Exp. Child Psychol. 116, 86–95. 10.1016/j.jecp.2013.02.007 23660178

[B82] Okumura Y. Kanakogi Y. Kobayashi T. Itakura S. (2017). Individual differences in object-processing explain the relationship between early gaze-following and later language development. Cognition 166, 418–424. 10.1016/j.cognition.2017.06.005 28624708

[B83] Okumura Y. Kobayashi T. Itakura S. (2016). Eye contact affects object representation in 9-month-old infants. PLoS ONE 11:e0165145. 10.1371/journal.pone.0165145 27776155 PMC5077079

[B84] Parise E. Csibra G. (2013). Neural responses to multimodal ostensive signals in 5-month-old infants. PLoS ONE 8:e72360. 10.1371/journal.pone.0072360 23977289 PMC3747163

[B85] Parise E. Handl A. Palumbo L. Friederici A. D. (2011). Influence of eye gaze on spoken word processing: an ERP study with infants. Child Dev. 82, 842–853. 10.1111/j.1467-8624.2010.01573.x 21410929

[B86] Parise E. Reid V. M. Stets M. Striano T. (2008). Direct eye contact influences the neural processing of objects in 5-month-old infants. Soc. Neurosci. 3, 141–150. 10.1080/17470910701865458 18633855

[B87] Paulus M. Fikkert P. (2014). Conflicting social cues: fourteen- and 24-month-old infants' reliance on gaze and pointing cues in word learning. J. Cogn. Dev. 15, 43–59. 10.1080/15248372.2012.698435

[B88] Reid V. M. Striano T. (2005). Adult gaze influences infant attention and object processing: Implications for cognitive neuroscience. Eur. J. Neurosci. 21, 1763–1766. 10.1111/j.1460-9568.2005.03986.x 15845105

[B89] Reid V. M. Striano T. Kaufman J. Johnson M. H. (2004). Eye gaze cueing facilitates neural processing of objects in 4-month-old infants. Neuroreport 15, 2553–2555. 10.1097/00001756-200411150-00025 15538194

[B90] Richards J. E. Reynolds G. D. Courage M. L. (2010). The neural bases of infant attention. Curr. Dir. Psychol. Sci. 19, 41–46. 10.1177/0963721409360003 20445766 PMC2863352

[B91] Senju A. Csibra G. (2008). Gaze following in human infants depends on communicative signals. Curr. Biol. 18, 668–671. 10.1016/j.cub.2008.03.059 18439827

[B92] Senju A. Johnson M. H. Csibra G. (2006). The development and neural basis of referential gaze perception. Soc. Neurosci. 1, 220–234. 10.1080/17470910600989797 18633789

[B93] Striano T. Chen X. Cleveland A. Bradshaw S. (2006a). Joint attention social cues influence infant learning. Eur. J. Dev. Psychol. 3, 289–299. 10.1080/17405620600879779

[B94] Striano T. Reid V. M. Hoehl S. (2006b). Neural mechanisms of joint attention in infancy. Eur. J. Neurosci. 23, 2819–2823. 10.1111/j.1460-9568.2006.04822.x 16817886

[B95] Szufnarowska J. Rohlfing K. J. Fawcett C. Gredeback G. (2014). Is ostension any more than attention? Sci. Rep. 4:5304. 10.1038/srep05304 24931735 PMC4058873

[B96] Tenenbaum E. J. Sobel D. M. Sheinkopf S. J. Malle B. F. Morgan J. L. Shah R. J. (2015). Attention to the mouth and gaze following in infancy predict language development. J. Child Lang. 42, 1173–1190. 10.1017/S0305000914000725 25403090 PMC8281329

[B97] Theuring C. Gredebäck G. Hauf P. (2007). Object processing during a joint gaze following task. Eur. J. Dev. Psychol. 4, 65–79. 10.1080/17405620601051246

[B98] Urakawa S. Takamoto K. Ishikawa A. Ono T. Nishijo H. (2015). Selective medial prefrontal cortex responses during live mutual gaze interactions in human infants: an fNIRS study. Brain Topogr. 28, 691–701. 10.1007/s10548-014-0414-2 25367848

[B99] Vecera S. P. Johnson M. H. (1995). Gaze detection and the cortical processing of faces: evidence from infants and adults. Vis. Cogn. 2, 59–87. 10.1080/13506289508401722

[B100] Wahl S. Marinović V. Träuble B. (2019). Gaze cues of isolated eyes facilitate the encoding and further processing of objects in 4-month-old infants. Dev. Cogn. Neurosci. 36:100621. 10.1016/j.dcn.2019.100621 30716584 PMC6969213

[B101] Wahl S. Michel C. Pauen S. Hoehl S. (2013). Head and eye movements affect object processing in 4-month-old infants more than an artificial orientation cue. Br. J. Dev. Psychol. 31, 212–230. 10.1111/bjdp.12001 23659892

[B102] Ward L. M. (2003). Synchronous neural oscillations and cognitive processes. Trends Cogn. Sci. 7, 553–559. 10.1016/j.tics.2003.10.012 14643372

[B103] Werker J. F. Cohen L. B. Lloyd V. L. Casasola M. Stager C. L. (1998). Acquisition of word–object associations by 14-month-old infants. Dev. Psychol. 34, 1289–1309. 10.1037/0012-1649.34.6.1289 9823513

[B104] Woodward A. L. (2003). Infants' developing understanding of the link between looker and object. Dev. Sci. 6, 297–311. 10.1111/1467-7687.00286

[B105] Woodward A. L. Markman E. M. Fitzsimmons C. M. (1994). Rapid word learning in 13- and 18-month-olds. Dev. Psychol. 30, 553–566. 10.1037/0012-1649.30.4.553

[B106] Wu R. Gopnik A. Richardson D. C. Kirkham N. Z. (2011). Infants learn about objects from statistics and people. Dev. Psychol. 47, 1220–1229. 10.1037/a0024023 21668098

[B107] Wu R. Kirkham N. Z. (2010). No two cues are alike: depth of learning during infancy is dependent on what orients attention. J. Exp. Child Psychol. 107, 118–136. 10.1016/j.jecp.2010.04.014 20627258

[B108] Wu R. Tummeltshammer K. S. Gliga T. Kirkham N. Z. (2014). Ostensive signals support learning from novel attention cues during infancy. Front. Psychol. 5:251. 10.3389/fpsyg.2014.00251 24723902 PMC3971204

[B109] Yoon J. M. D. Johnson M. H. Csibra G. (2008). Communication-induced memory biases in preverbal infants. Proc. Natl. Acad. Sci. U.S.A. 105, 13690–13695. 10.1073/pnas.0804388105 18757762 PMC2533251

[B110] Yu C. Smith L. B. (2017). Hand-eye coordination predicts joint attention. Child Dev. 88, 2060–2078. 10.1111/cdev.12730 28186339 PMC6894731

[B111] Yu C. Suanda S. H. Smith L. B. (2019). Infant sustained attention but not joint attention to objects at 9 months predicts vocabulary at 12 and 15 months. Dev. Sci. 22:e12735. 10.1111/desc.12735 30255968 PMC6918481

[B112] Yurovsky D. Frank M. C. (2017). Beyond naive cue combination: salience and social cues in early word learning. Dev. Sci. 20:e12349. 10.1111/desc.12349 26575408 PMC4870162

