# Supplementary material for: Do the Eyes Have It? A Systematic Review on the Role of Eye Gaze in Infant Language Development
Source: Front Psychol. 2021 Jan 8;11:589096. doi: 10.3389/fpsyg.2020.589096 (PMC7874056; doi:10.3389/fpsyg.2020.589096)
Supplement: Supplementary file 1 [file Data_Sheet_1.PDF]

Supplementary Table Çetinçelik et al. (2020)

	Section	Author (year)	*N*	Infant age (months)	Method	Main relevant findings
**1.**	**Vocabulary development**	**Beuker et al. (2013)**	23	8 to 24	Behavioral; longitudinal study	JA skills emerged between 8-15 months. Gaze following predicted receptive vocabulary growth, directing attention predicted receptive and expressive vocabulary growth.
**2.**		**Brooks & Meltzoff (2005)**	32; 32; 32	9, 10, 11, 14, 18	Behavioral; longitudinal study	Gaze following behavior plus vocalizations at 10-11 months predicted 14- and 18-month receptive vocabulary.
**3.**		**Brooks & Meltzoff (2008)**	32	10-11, 14, 18, 24	Behavioral; longitudinal study	Gaze following behavior at 10-11 months predicted vocabulary growth through the 10-24-month period, controlling for age and maternal education. Adding pointing to the model strengthened the model.
**4.**		**Brooks & Meltzoff (2015)**	32	10-11, 30, 54	Behavioral; longitudinal study	Gaze following scores at 10-11 months predicted children's productive vocabulary at 30 months (more significantly so for mental-state terms).
**5.**		**Carpenter et al. (1998)**	24	9-15	Behavioral; longitudinal study	Infants' joint engagement with their caregivers at 11, 12, and 13 months predicted their receptive vocabulary through the 9-15-month period.
**6.**		**Charman et al. (2000)**	13	20, 44	Behavioral; longitudinal study	Gaze switches between an adult and toy at 22 months were not significantly associated with 44-month receptive or expressive vocabulary; although it predicted later Theory of Mind abilities.
**7.**		**D'Odorico et al. (1997)**	13	4, 6, 8, 10, 12, 16, 20	Behavioral; longitudinal study	Gaze and vocalizations predicted later vocabulary growth.
**8.**		**De Schuymer et al. (2011)**	35	6, 9, 14, 30	Behavioral; longitudinal study	Infants' gaze following in triadic interactions at 9 and 14 months predicted receptive and expressive vocabulary at 30 months.

Studies marked with (*) are also relevant for section 3.1.5 "Why does gaze facilitate learning?"

Section	Author (year)	*N*	Infant age (months)	Method	Main relevant findings
**9.**	**Markus et al. (2000)**	21	12, 18, 21, 24	Behavioral; longitudinal study	Responding to joint attention at 12 months was linked to expressive language at 18, 21 and 24 months.
**10.**	**Morales et al. (2000a)**	22	6, 8, 10, 12, 15, 18, 21, 24, 30	Behavioral; longitudinal study	Responding to joint attention at 6, 8, 10, 12, and 18 months was positively related to individual differences in receptive and expressive vocabulary development.
**11.**	**Morales et al. (2000b)**	52	6, 12	Behavioral; longitudinal study	Responding to joint attention at 6 months was positively related to receptive vocabulary at 12 months.
**12.**	**Morales et al. (1998)**	21	6	Behavioral; longitudinal study;	Language follow-up at 12, 18, 21, 24 months. Gaze following skills at 6 months were positively related to receptive vocabulary at 12 months, and expressive vocabulary at 18 and 24 months.
**13.**	**Mundy et al. (2007)**	95	9-18, 24	Behavioral; longitudinal study	Responding to joint attention at 9 and 12 months (and initiating joint attention at 18 months) predicted (composite) language scores at 24 months.
**14.**	**Mundy et al. (2003b)**	29	14, 18, 24	EEG; longitudinal study	EEG coherence and joint attention were longitudinally associated with individual differences in language outcomes.
**15.**	**Mundy & Gomes (1998)**	24	14-17	Behavioral; longitudinal study	Responding to joint attention correlated significantly with expressive and receptive vocabulary at the time of follow-up.
**16.**	**Tenenbaum et al. (2015)**	61	12, 18, 24	Behavioral; longitudinal study	Gaze following and attention to the speaker’s mouth at 12 months predicted expressive vocabulary at 18 and 24 months.
**17.**	**Yu et al. (2019)**	26	9	Behavioral	Infants’ sustained attention, within the context of joint attention, during infant-caregiver free play episodes, predicted vocabulary size at 12 and 15 months.

Studies marked with (*) are also relevant for section 3.1.5 “Why does gaze facilitate learning?”

	Section	Author (year)	*N*	Infant age (months)	Method	Main relevant findings
**18.**	**Word-object mapping**	**Barry-Anwar et al. (2017)**	49	12-18	Behavioral	Infants who followed their caregiver's gaze more formed stronger word-object associations. Infants' followed the gaze of a stranger and caregiver equally well but learned labels only in the caregiver condition.
**19.**		**Gogate et al. (2006)**	24	6-8	Behavioral	Infants who alternated gaze between caregiver and object most frequently during play episodes learned word-object mappings.
**20.**		**Graham et al. (2010)**	66 (Exp 1A); 42 (Exp 1B); 36 (Exp 2)	24	Behavioral	When eye gaze and mutual exclusivity provided inconsistent information, children relied on mutual exclusivity to form word-object mappings. Learning was optimized when both cues converged.
**21.**		**Graham et al. (2011)**	30 (Exp 1); 46 (Exp 2)	24	Behavioral	Infants were able to form word-object mappings when objects are highlighted with non-social attentional cues; however, when cued by eye gaze, they formed word-object, not word-location mappings, suggesting that eye gaze direction may mark intentionality for 24-month-old infants.
**22.**		**Hirotani et al. (2009)**	23	18-21	EEG	A late negativity (possibly reflecting impaired semantic integration) was observed for incongruent word-object pairs, only when word-object pairs were previously taught in a joint attentional context.
**23.**		**Hollich et al. (2000)**	32; 32; 32	12; 19; 24	Behavioral	24-month-old infants follow adult's gaze direction to map words to objects, rather than salient but non-referential cues.
**24.**		**Houston-Price et al. (2006)**	27 (Exp 1); 30 (Exp 2); 30 (Exp 3); 32 (Exp 4)	15	Behavioral	Infants used gaze cues to match labels to objects under tightly controlled conditions, such as a video of a face turning to the target when labels are provided over loudspeakers. They also used the non-social salience cue to form word-object mappings.
**25.**		**Matatyaho & Gogate (2008)**	24	6-8	Behavioral	Infants' gaze alternation skills and mothers' naming behaviors predicted infants' word-object mappings.

Studies marked with (*) are also relevant for section 3.1.5 "Why does gaze facilitate learning?"

	Section	Author (year)	*N*	Infant age (months)	Method	Main relevant findings
**26.**		**Moore et al. (1999)**	75; 75	18; 24	Behavioral	24-month-olds, but not 18-month-olds were guided by the adult's gaze direction, even when saliency of the other object was higher. Eighteen-month-old infants only formed word-object mappings when referential and salience cues converged.
**27.**		**O'Connell et al. (2009)**	26 (Exp 1); 25 (Exp 2); 28 (Exp 3)	18	Behavioral	Infants followed the gaze of a human and non-human (robot) agent, but only learned word-object mappings when the interlocutor was human.
**28.**		**Paulus & Fikkert (2014)**	16; 16	14; 24	Behavioral	14-month-olds relied on the speaker's eye gaze direction when learning word-object mappings, whereas 24-month-olds (and adults) relied more on pointing cues.
**29.**		**Yurovsky & Frank (2015)**	Exp 1: 137 Exp 2: 104 (see paper for *N*s)	1-4 years	Behavioral	Children's word learning is supported by social cues, but their sensitivity to social cues develop gradually through the development of attention, memory, and information processing capacities.
**30.**	**Object processing**	**Barry et al. (2015)**	55	9	Behavioral	Both social and non-social cues guide infants' attention to visual statistical regularities. Although the social cue was more effective in the familiarization phase, it did not lead to better learning.
**31.**		**Cleveland et al. (2007)**	30; 30	4; 9	Behavioral	Adult's use of joint attention during object viewing facilitated 9-month-olds' (but not 4-month-olds) object processing.
**32.**		**Cleveland & Striano (2007)**	16; 15	5; 7	Behavioral	Adult's use of joint attention during object viewing facilitated 7-month-olds' (but not 5-month-olds) object processing.
**33.**		**Hoehl et al. (2014a)***	24	9	EEG	When viewing objects together with an adult, infants showed desynchronization of alpha activity when the adult offered eye contact, but not when the adult looked only at the object.
**34.**		**Hoehl et al. (2008a)***	17	4	EEG	Infants had enhanced PSW and more negative Nc for object-directed compared to object-averted gaze.

Studies marked with (*) are also relevant for section 3.1.5 "Why does gaze facilitate learning?"

Section	Author (year)	*N*	Infant age (months)	Method	Main relevant findings
**35.**	**Hoehl et al. (2012)***	16	4	EEG	Caregiver's gaze had a greater effect on infants' object processing compared to a stranger's: infants' PSWs were enhanced for uncued objects compared to cued ones only when the caregiver provided the object-directed gaze cues.
**36.**	**Hoehl et al. (2014b)***	46	4	EEG	Infants' attention to objects and object processing can be cued by both the head orientation and eye gaze shifts.
**37.**	**Hoehl et al. (2008b)***	15	3	EEG	Infants had enhanced Ncs in response to objects when the adult gazed at the object with a frightened expression.
**38.**	**Hutman et al. (2016)***	11	20	EEG	Following object presentation in interactions with joint engagement, infants had larger Pb and smaller Nc to objects compared to the non-joint engagement condition, indicating more familiarity with objects when they were presented in a joint attentional episode.
**39.**	**Kopp & Lindenberger (2011)***	28	9	EEG	The degree of joint attention when viewing objects modulated the PSW amplitude immediately after the learning phase, and the amplitude of the Pb component one week after the initial test, indicating effects of joint attention on long-term memory processing.
**40.**	**Kopp & Lindenberger (2012)**	30	4	EEG	The PSW to objects did not change as a function of joint attention during object processing, but the Pb component was modulated by joint attention in the delayed recognition test after one week. Infant's gazing frequency at the experimenter modulated the Pb response.
**41.**	**Michel et al. (2017)***	42	4	Behavioral	Moving schematic images of isolated eyes to an object functioned as a gaze cue and led to enhanced object processing.
**42.**	**Michel et al. (2019)***	18	4	EEG	Eye gaze cues led to enhanced PSW for uncued objects compared to cued ones, whereas non-social attentional

Studies marked with (*) are also relevant for section 3.1.5 "Why does gaze facilitate learning?"

Section	Author (year)	*N*	Infant age (months)	Method	Main relevant findings
					cues did not result in differential neural activity in relation to objects.
**43.**	**Okumura et al. (2013a)**	32 (Exp 1); 16 (Exp 2); 16 (Exp 3)	12	Behavioral	Although infants followed both the human and robot's gaze, only human gaze facilitated processing of the cued object.
**44.**	**Okumura et al. (2013b)**	32; 32	10; 12	Behavioral	Both groups of infants followed gaze of a human and robot. However, only 12-month-olds predicted the location of an object, only when cued by human gaze.
**45.**	**Okumura et al. (2016)**	28	9	Behavioral	Adult's use of eye contact during social interactions involving an object altered the kind of information infants learned about the object. When the interaction involved mutual eye gaze, infants processed the object's identity but not its location.
**46.**	**Okumura et al. (2017)**	37	9	Behavioral	Infants who followed gaze longer processed objects more efficiently at 9 months, and their larger expressive vocabulary size was larger at 18 months. Object processing performance mediated the relationship between early gaze following abilities and later vocabulary size.
**47.**	**Parise et al. (2008)***	15	5	EEG	When the experimenter provided direct eye contact to the infants when viewing objects together with the infants, infants' ERP responses showed a more negative Nc in response to the object, compared to a no-eye-contact condition.
**48.**	**Reid & Striano (2005)***	22 (Exp 1); 19 (Exp 2)	4	Behavioral	After familiarization with object-directed gaze, infants displayed a novelty preference for the uncued object, indicating familiarity with the cued object.
**49.**	**Reid et al. (2004)***	12	4	EEG	Infants had enhanced Slow Wave responses to uncued objects compared to cued ones, indicating familiarity with the cued object.

Studies marked with (*) are also relevant for section 3.1.5 "Why does gaze facilitate learning?"

Section	Author (year)	*N*	Infant age (months)	Method	Main relevant findings
**50.**	**Striano et al. (2006a)***	24	9; 12	Behavioral	9-month-old infants looked longer at a novel toy after a period of interaction with the adult experimenter involving joint attention, but not in the object only condition. 12-month-olds' looking times did not differ between the two conditions.
**51.**	**Striano et al. (2006b)***	15	9	EEG	An enhanced Nc was observed for objects presented in periods of interaction involving joint attention, with direct eye contact.
**52.**	**Theuring et al. (2007)**	16	12	Behavioral	A novelty preference for the uncued object was observed compared to the cued object, for the first test trial.
**53.**	**Wahl et al. (2019)**	17 (Exp 1); 19 (Exp 2)	4	EEG	Gaze cues of isolated eyes functioned the same way as the full-face gaze cues. An enhanced PSW was observed for object-directed gaze, and enhanced Nc amplitude and increased looking times were observed for uncued objects.
**54.**	**Wahl et al. (2013)***	22 (Exp 1); 18 (Exp 2)	4	EEG	Social gaze cues enhanced object processing (increased Nc and longer looking times to uncued objects) whereas a car instead of the human head did not lead to significant differences in infants' neural processing of objects.
**55.**	**Wu et al. (2011)**	18 (Exp 1); 18 (Exp 2); 17 (Exp 3); 18 (Exp 4)	9	Behavioral	Infants use feature co-occurrences when learning about objects, and eye gaze cueing (face turning towards the object) help them in forming those statistical inferences, even when distractors are present.
**56.**	**Wu et al. (2014)***	16 (Exp 1); 16 (Exp 2); 17 (Exp 3)	8	Behavioral	Infants learned where multimodal events occurred best when an ostensive social cue preceded the non-social attentional cue.
**57.**	**Yoon et al. (2008)**	24	9	Behavioral	Infants learned about the object's identity, but not location, when they interacted with the adult experimenter in a communicative context. When the interaction was non-communicative, they retained information about location, but not identity.

Studies marked with (*) are also relevant for section 3.1.5 "Why does gaze facilitate learning?"

	Section	Author (year)	*N*	Infant age (months)	Method	Main relevant findings
**58.**	**Speech processing**	**Conboy et al. (2015)**	21	9.5-10.5	EEG	Infants' gaze shifts between object and interlocutor during foreign language exposure sessions at 9.5-10.5 months predicted their foreign language phoneme discrimination (assessed by their ERPs) at 11 months.
**59.**		**Lloyd-Fox et al. (2015)**	24 (Exp 1); 24 (Exp 2)	6	fNIRS; natural interaction	Infant-directed speech in combination with direct eye gaze led to greater activation compared to infant-directed speech alone in the inferior frontal, anterior temporal, and temporo-parietal regions.
**60.**		**Parise et al. (2011)**	15 (Exp 1); 15 (Exp 2)	4-5	EEG	Enhanced Negative Component and late Slow Wave for backwards-spoken words when presented with direct gaze, compared to averted gaze. Experiment 2 found the same results for object-directed gaze.
**61.**	**Why does eye gaze facilitate learning?**	**Csibra & Volein (2008)**	16; 16	8; 12	Behavioral	Infants at both ages looked longer at a location cued by the experimenter's gaze if it was empty after the barriers were removed, indicating an understanding of referential gaze information.
**62.**		**de Bordes et al. (2013)**	56	20	Behavioral	Infants followed gaze after ostensive (eye contact) and non-ostensive (colorful image covering the eyes) interaction.
**63.**		**Farroni et al. (2000)**	15 (Exp 1); 17 (Exp 2); 30 (Exp 3)	4-5	Behavioral	The perceived motion of the pupils directs infants' attention to follow the interlocutor's gaze.
**64.**		**Gredebäck et al. (2010)**	40	2, 4, 6, 8	Behavioral, longitudinal	Gaze following abilities emerge between 2-4 months and become stable by 6-8 months. Infants showed a stranger preference when gaze following between 4-6 months, suggesting that gaze following does not depend on reinforcement learning alone.
**65.**		**Gredebäck et al. (2018)**	94	6	Behavioral	Infants followed gaze equally well after ostensive and non-ostensive interactions.

Studies marked with (*) are also relevant for section 3.1.5 "Why does gaze facilitate learning?"

Section	Author (year)	*N*	Infant age (months)	Method	Main relevant findings
**66.**	**Grossmann et al. (2007)**	12	4	EEG	Direct gaze resulted in stronger early gamma activity in occipital regions, as well as a late-induced gamma burst in prefrontal regions, compared to averted gaze.
**67.**	**Grossmann et al. (2008)**	12 (Exp 1); 12 (Exp 2)	4	fNIRS (Exp 1); EEG (Exp 2)	Mutual eye gaze resulted in adult-like activation in superior posterior temporal cortex and right frontopolar cortex. Mutual gaze led to similar activation as mutual gaze with accompanying smile or eyebrow raise, suggesting its interpretation as a communicative signal.
**68.**	**Johnson et al. (2007)**	20	9	Behavioral	9-month-olds interpreted an adult's gaze and head turns toward an object when the action was embedded in multiple actions, showing that they may understand the goal-directed nature of gaze shifts.
**69.**	**Michel et al. (2015)**	14;16;16;12	2; 4; 5; 9	EEG	4- and 9-month-old infants had more alpha desynchronization in response to object-directed gaze. 5-month-old showed more theta synchronization in response to object-averted gaze. 2-month-olds' neural activity did not differ for object-directed and object-averted conditions.
**70.**	**Moll & Tomasello (2004)**	39 (Exp 1); 32 (Exp 2)	18	Behavioral	12- and 18-month-old infants followed experimenter's gaze behind barriers where an object is hidden, suggesting an understanding of referential information conveyed by gaze.
**71.**	**Moore et al. (1997)**	45 (Exp 1A); 15 (Exp 1B); 46 (Exp 2A); 15 (Exp 2B)	9	Behavioral	Infants who did not follow gaze learned to follow gaze after reinforcement, suggesting that gaze following might depend on reinforcement learning.
**72.**	**Parise & Csibra (2013)**	18 (Exp 1); 18 (Exp 2)	5	EEG	Eye gaze cues and infant-directed speech elicited overlapping cortical activity. The combination of the two signals did not result in greater activation.

Studies marked with (*) are also relevant for section 3.1.5 "Why does gaze facilitate learning?"

Section	Author (year)	*N*	Infant age (months)	Method	Main relevant findings
**73.**	**Senju & Csibra (2008)**	14 (Exp 1); 12 (Exp 2); 12 (Exp 3); 12 (Exp 4)	9	Behavioral	Infants attend more to object-directed gaze. Eye contact triggers the object-gaze congruency effect.
**74.**	**Senju et al. (2006)**	10	9	EEG	Infants had similar ERP responses with adults (N290; N330 for adults) to object-averted gaze shifts, as well as an anterior N200, which was higher in amplitude for object-congruent gaze direction.
**75.**	**Szufnarowska et al. (2014)**	22	6	Behavioral	Infants followed the adult's gaze after both ostensive and non-ostensive cues.
**76.**	**Urakawa et al. (2015)**	11	7	NIRS	During social play, adult's mutual eye contact with the infant resulted in longer infant fixations at the adult's eyes, as well as increased activity in mPFC.
**77.**	**Wu & Kirkham (2010)**	29 (Exp 1); 60 (Exp 2); 44 (Exp 3)	4; 8 (Exp 1); 8 (Exp 2 and 3)	Behavioral	8-month-olds specifically learned audiovisual events after being presented with social cues, whereas 4-month-olds showed general spatial learning. 8-month-olds did not display learning when non-social attentional signals cued the events.

Studies marked with (*) are also relevant for section 3.1.5 "Why does gaze facilitate learning?"
